# ASAP2 interrupts c-MET-CIN85 interaction to sustain HGF/c-MET-induced malignant potentials in hepatocellular carcinoma

**DOI:** 10.1186/s40164-023-00393-3

**Published:** 2023-04-15

**Authors:** Xiao-Lu Ma, Yan-Yan Nie, Su-Hong Xie, Hui Zheng, Ying Tong, Yan-Chun Wang, Tian-Qing Yan, Xin Meng, Jia-Zhen Cao, Wei-Guo Tang, Lin Guo, Ren-Quan Lu

**Affiliations:** 1 Department of Clinical Laboratory, Shanghai Cancer Center, Fudan University, Shanghai, 200032 China; 2 Department of Oncology, Shanghai Medical School, Fudan University, Shanghai, 200032 China; 3 Shanghai Lab. Animal Research Center, Shanghai, 201203 China; 4 Liver Cancer Institute, Zhongshan Hospital, Fudan University, Shanghai, 200032 China; 5 Department of Hepatobiliary and Pancreatic Surgery, Minhang Hospital, Fudan University, Shanghai, 201100 China

**Keywords:** Hepatocellular carcinoma, Epithelial-mesenchymal transition, c-MET signaling, ASAP2, CIN85

## Abstract

**Background:**

Sustained activation of hepatocyte growth factor (HGF)/c-MET signaling is a major driver of hepatocellular carcinoma (HCC) progression, but underlying mechanism is unclear. ArfGAP With SH3 Domain, Ankyrin Repeat And PH Domain 2 (ASAP2) can reportedly activate GTPases and promote receptor tyrosine kinase signaling. However, the exact role of ASAP2 in HCC, especially for c-MET activation, also remains elusive.

**Methods:**

ASAP2 expression levels in HCC tissues and cells were quantified using qRT-PCR, western blot (WB) analysis, and immunohistochemistry staining. Cell counting kit-8 (CCK-8) and colony formation assays were performed to evaluate cell proliferation rates. Flow cytometry assays were conducted to assess apoptosis rates. Wound healing and Transwell assays were performed to determine cell migration and invasion capacities. Epithelial-mesenchymal transition (EMT)-related marker expression levels were also examined. Subcutaneous implantation and tail vein injection models were applied for in vivo growth and metastasis evaluations, respectively. Bioinformatics analyses of The Cancer Genome Atlas and STRING datasets were performed to explore ASAP2 downstream signaling. Co-immunoprecipitation and Cycloheximide chasing experiments were performed to assess protein–protein interactions and protein half-life, respectively.

**Results:**

ASAP2 had higher expression levels in HCC tissues than in normal liver, and also predicted poor prognosis. Knocking down ASAP2 significantly impaired cell proliferation, migration, and invasion capacities, but promoted apoptosis in HCC cells in vitro. However, overexpression of ASAP2 achieved the opposite effects. In vivo experiments confirmed that ASAP2 could promote HCC cell growth and facilitate lung metastasis. Interestingly, ASAP2 was essential for triggering EMT. Gene Set Enrichment Analysis demonstrated that c-MET signaling was greatly enriched in ASAP2-high HCC cases. Additionally, c-MET signaling activity was significantly decreased following ASAP knockdown, evidenced by reduced c-MET, p-AKT, and p-ERK1/2 protein levels. Importantly, ASAP2 knockdown effectively attenuated HGF/c-MET signaling-induced malignant phenotypes. c-MET and ASAP2 expression levels were positively correlated in our cohort. Mechanistically, ASAP2 can directly bind to CIN85, thereby disrupting its interaction with c-MET, and can thus antagonize CIN85-induced c-MET internalization and lysosome-mediated degradation. Notably, knocking down CIN85 can rescue the observed inhibitory effects caused by ASAP2 knockdown.

**Conclusions:**

This study highlights the importance of ASAP2 in sustaining c-MET signaling, which can facilitate HCC progression.

**Supplementary Information:**

The online version contains supplementary material available at 10.1186/s40164-023-00393-3.

## Introduction

Hepatocellular carcinoma (HCC) is currently the most common solid tumor worldwide. Its incidence has continued to escalate in recent years, especially in China, posing a great challenge to public health [1, 2]. Even with the clinical advances that have been achieved in recent years, including systemic immunotherapy, the overall survival (OS) rate remains unsatisfactory with over 50% of patients dying within 5 years of initial therapy [3]. Accumulating evidence has demonstrated that the main contributor to the mortality rate is the rapid disease progression from tumor recurrence [4, 5]. Critically, aggressive invasiveness is widely considered as the main driving force for HCC metastasis and recurrence [6]. Thus, a better understanding of enhanced HCC invasiveness may provide novel insights into preventing HCC progression.

Aberrant signaling pathway activation is a hallmark and core mechanism that mediates HCC metastasis [7]. Notably, hepatocyte growth factor (HGF)-induced c-MET signaling activation was identified as the crucial molecular event for triggering invasion and recurrence in HCC [8, 9]. As a receptor tyrosine kinase expressed on the cell membrane, c-MET is activated after sensing extracellular HGF by forming a homodimer and autophosphorylation [10]. Activated c-MET can recruit and phosphorylate PI3K/AKT and MAPK/ERK kinases to support increased HCC cell invasion [11, 12]. Importantly, c-MET signaling was also identified as essential for triggering the epithelial-mesenchymal transition (EMT) [13], a pathological process where cells gain a mesenchymal-like phenotype with loss of epithelial-like properties. EMT plays a vital role in promoting HCC recurrence [14]. Generally, c-MET signaling is strictly controlled in normal cells, where activated c-MET ultimately interacts with Cbl Proto-Oncogene (c-CBL), an E3-ligase, and undergoes ubiquitination followed by intracellular trafficking and lysosome-mediated degradation [15, 16]. However, this control mechanism does not function in HCC cells, but the underlying molecular details of this remain elusive.

ArfGAP With SH3 Domain, Ankyrin Repeat And PH Domain 2 (ASAP2), a member of the conventional ArfGAP family of proteins, reportedly exerts dramatic functions in tumor proliferation and mobility [17]. Moreover, high ASAP2 expression levels were associated with pancreatic cancer recurrence. Importantly, ASAP2 was identified as one hub gene for tumoral co-expression network construction. These discoveries strongly suggested that ASAP2 is a crucial driver gene in cancer development. However, ASAP2 expression patterns in HCC and the association with tumor recurrence are still unclear, and the underlying mechanism by which ASAP2 can mediate HCC invasion and recurrence is also currently not well understood. Hence, in present study, we aimed to explore ASAP2 expression in HCC and further demonstrate its biological function and prognostic value in this disease. Moreover, we report an association between ASAP2 and c-MET signaling-induced EMT and reveal how ASAP2 contributes to the long-term activation of c-MET signaling in HCC.

## Materials and methods

### Patients and follow-up

Two independent cohorts of HCC patients who received curative resection were enrolled: cohort 1 (*n* = 19), fresh cancerous tissues were collected from January 2021 to December 2021 from Fudan University Shanghai Cancer Center (FUSCC) to assess ASAP2 expression levels; cohort 2 (*n* = 49), samples were collected from January 2017 to December 2017 from FUSCC and were used for immunohistochemistry (IHC). This study was approved by the FUSCC Research Ethics Committee. All individuals enrolled provided informed consent for inclusion of their tissue in the present study. The inclusion criteria were as follows: (1) Precise HCC diagnoses were according to histopathological examinations following the American Association for Study of Liver Disease guidelines [18]; (2) no prior anti-cancer therapy history was recorded; (3) received curative resection, which was defined as complete removal of all tumor nodules, without any loci found on the incision surface during histological determination; (4) availability of appropriate paraffin embedded HCC or frozen tissues; and (5) complete clinicopathological and follow-up data [18]. Follow-up was performed as previously described [19]. Follow-up ended in March 2022. Recurrence-free survival (RFS) was defined as the interval between the time of resection to the time of finding any sign of intrahepatic tumoral nodule, and OS was defined as the interval between the time of resection to the time of death or the time of the end of follow-up period.

### Cell lines and in vivo tumor growth evaluation and pulmonary metastasis determinations

Human cell lines MIHA, PLC/PRF/5, Huh7, and Hep3B were obtained from The Liver Cancer Institute (Fudan University, Shanghai, China); HCC cell lines SNU182 and SNU387 were purchased from Shanghai Institute of Cell Biology (Chinese Academy of Sciences). All cells used in the present study were maintained in DMEM (Gibco, Grand Island, NY, USA) supplemented with 10% fetal bovine serum and 1% antibiotic (Gibco) at 37 °C with 5% CO_2_.

For in vivo growth evaluation, SNU182-shControl, SNU182-shASAP2, SNU387-shControl, and SNU387-shASAP2 cells were suspended in a 1:1 volumetric mixture of 100 μL serum-free DMEM (Gibco) and Matrigel (BD Biosciences, Franklin Lakes, NJ, USA). Thereafter, the cell mixtures were subcutaneously injected into the upper flank of 4-week-old BALB/c nude mice (six mice/group). After 4 weeks of feeding, the nude mice were sacrificed by pentobarbital overdose to obtain tumor tissues. Tumor dimensions were measured, and tumor volumes (mm^3^) were calculated according to the following formula: V = (ab^2^)/2, where a and b are the largest and smallest tumor diameters, respectively. Establishment of animal models and animal assays were approved by The Research Ethical Committee of FUSCC. For lung metastasis evaluation, 4-to-6-week-old BALB/c nude mice were selected and randomly assigned to two groups (six mice per group). Thereafter, mice were injected with 5 × 10^5^ of the SNU182-shControl or SNU182-shASAP2 cells via the tail vein. After 6 weeks, the lung tissues were separated and embedded in paraffin, then stained with hematoxylin and eosin to observe metastatic loci under a microscope as described previously [20]. The occurrence of tumor lesions was recorded as positive for lung metastases, and the incidence of the lung metastases was calculated.

### RNA extraction and qRT-PCR

Total RNA was extracted using a RNeasy mini kit (Qiagen, Germany) according to the manufacturer’s instructions. Extracted RNA was quantified using a NanoDrop 2000 (Thermo Fisher, USA), and cDNA was synthesized using the SuperScript IV First-Strand Synthesis System kit (Invitrogen, USA) according to the manufacturer’s instructions. Quantitative real time-PCR (qRT-PCR) was performed with TB Green Fast qPCR Mix (Takara, China) using a DX-II PCR instrument (ABI, USA). The quantities of target gene mRNAs were examined relative to an internal control using the ΔCq method. PCR conditions were as follows: 2 min at 95 °C, followed by 40 cycles of 95 °C for 20 s and 60 °C for 60 s. β-Actin was used as an internal control. Primers used in the present study are listed in Additional file 2: Table S1. All experiments were conducted in triplicate.

### Protein extraction and western blotting assays

Western blotting assays were conducted as previously reported [6]. Briefly, total intracellular protein was extracted using RIPA lysis buffer (Beyotime, Nantong, China) supplemented with protease inhibitor cocktail (Beyotime) and 0.1 mM PMSF (Beyotime) in accordance with the manufacturer’s instructions. Thereafter, protein concentrations were assessed using the Bicinchoninic Acid (BCA) Assay Kit (Beyotime). Equal quantities of protein lysates were resolved by sodium dodecyl sulfate–polyacrylamide gel electrophoresis (SDS-PAGE), followed by transfer to polyvinylidene fluoride (PVDF) membranes (0.45 μm, Burlington, Millipore, MA, USA). Membranes were blocked with a rapid block buffer (Beyotime, China). Then, membranes were incubated with specific primary antibodies targeting the indicated proteins overnight at 4 °C. After removing unconjugated primary antibodies, membranes were further incubated with the appropriate horseradish peroxidase-conjugated secondary antibody. After washing five times with Tris-buffered saline supplemented with 0.1% Tween-20, immunoreactive proteins on the membrane were detected with the BeyoECL Star Kit (Beyotime). The primary antibodies used are listed as Additional file 2: Table S2.

### Plasmids, transduction, and small interfering RNA (siRNA) transfection

First, short hairpin RNAs (shRNAs) targeting specific regions of the indicated genes were cloned into the pLKO.1 vector, and the entire ASAP2 coding sequence (CDS) sequence was cloned into the pLenti-CMV-puro plasmid. Scramble shRNA and empty pLenti-CMV-puro vector were used as controls (shControl and empty vec), respectively. Lentivirus was produced in HEK293 cells transfected with the above pLKO.1 or pLenti-CMV-puro plasmids as previously described [21]. Virus supernatant was collected 72 h after transfection, and viral particles were precipitated by PEG-8000. To generate stable-transfected HCC cells, lentivirus stocks were applied to transduce SNU182, SNU387, and Hep3B cells using 8 mg/L polybrene. Then, cells were cultured in selective medium supplemented with 2.5 mg/L puromycin 48 h after virus transduction for 7 to 12 days. Target sequences for the specific genes are described in Additional file 2: Table S3. The siRNAs targeting CIN85 were purchased from Merdobio (Shanghai, China) and transfected into indicated HCC cell lines using Lipofectamine 3000 reagent (Gibco) according to the manufacturer’s instructions. Target sequences are also listed in Additional file 2: Table S3.

### Cell counting kit-8 (CCK-8) and colony formation assays

For CCK-8 assays, the indicated HCC cells were seeded at a density of 1000 cells per well in a 96-well plate. Seeded cells were allowed to adhere to the well by culturing for overnight as Day 0. Afterwards, 10 μL CCK-8 working solution (Dojindo, Japan) was added into each well at the indicated time points. Then, cells were incubated at 37 °C for 2 h. Finally, the optical density of each well was measured at 450 nm using a microplate reader (Thermo Fisher) to assess the cell viability. For colony formation assays, HCC cells were seeded at a density of 800 cells per well and cultured in complete DMEM containing 10% FBS and 1% antibiotics for 14 days. Surviving cell colonies were fixed with methanol and then stained with 0.1% crystal violet for further quantification of colonies.

### Transwell assays

Migration and invasion potentials were assessed in 24-well plates with Transwell chamber inserts (8 μm pore size; Millipore, USA) as previously reported [19]. Specifically, for invasion assays, Transwell chamber inserts were pre-coated with Matrigel (Millipore). For each assay, 5 × 10^4^ HCC cells were suspended in 100 μL serum-free DMEM in the upper chamber to form a single cell suspension, then 600 μL DMEM supplemented with 4% FBS was added into the lower chamber. Z-DEVD-FMK, a caspase-3 antagonist, was added to both the upper and lower chambers to prevent apoptosis. Moreover, cells were incubated for 24 h to avoid the impact of proliferation potential difference. After incubation, migrating or invading cells on the lower membrane surface were fixed with 4% paraformaldehyde, then stained with crystal violet (Beyotime). Images were acquired at 40 × magnification with a microscope (Olympus, Japan). All experiments were performed in triplicate.

### Wound healing assays

HCC cells were seeded at a density of 5 × 10^5^ cells per well in a 24-well plate and cultured in complete DMEM supplemented with 10% FBS and 1% antibiotics. Once the cells were 90% confluent, a scratch was made with a 200 μL sterile pipette tip and gently washed three times with PBS to remove exfoliated cells. Thereafter, fresh complete serum-free DMEM containing 1% antibiotics was added, and the cells were incubated at 37 °C. The wounds were examined at 0, 24, and 48 h after scratching and representative images were taken with a microscope. All experiments were performed in triplicate.

### Apoptosis assays

Apoptosis rates of HCC cells were quantified by flow cytometry analysis as previously described [6]. In brief, HCC cells given indicated treatments were harvested, then washed three times with ice cold PBS. Then, HCC cells were stained using FITC-conjugated Annexin-IV and Propidium Iodide (PI) (BD Biosciences, USA) according to the manufacturer’s instructions, followed by three washes with ice cold PBS. Stained cells were subjected to flow cytometry analysis and surviving cells were defined as dual-negative for FITC and PI.

### Regimens and treatments

For HGF stimulation, HGF was added into the culture medium at a concentration of 20 ng/mL. To inhibit intracellular protein synthesis, 50 μg/mL cycloheximide (CHX) was applied, and cells treated with CHX were harvested at the indicated time points for subsequent protein extraction and western blot assays. To prevent endogenous lysosomal c-MET degradation, HCC cells were pretreated with 100 nM bafilomycin A1 (BA) for 24 h prior to collection and further experiments. To prevent apoptosis of HCC cells in Transwell assays, 100 μM of Z-DEVD-FMK was added to both the upper and lower chambers.

### Cycloheximide (CHX) chasing assays

CHX chasing assays were performed to determine the half-life of c-MET under different treatments according to a previous study [6]. Briefly, indicated HCC cells were treated with CHX (50 μg/mL) for various times. Then, total protein was extracted and the c-MET protein levels were assessed by western blot analysis.

### Immunofluorescence staining

For detecting N-Cadherin and E-Cadherin expression, HCC cells were fixed with 4% paraformaldehyde and blocked with 5% bovine serum albumin. Next, HCC cells were stained with anti-N-Cadherin (1:100 CST) or anti-E-Cadherin (1:100 CST) at 4 °C overnight, followed by incubation with 594-conjugated anti-rabbit antibody (1:500, CST) for 2 h, and cells were counterstained with DAPI for observation under the microscope.

### Membrane protein enrichment and separation

Membrane protein fractions were separated using a Cell membrane protein and cytoplasmic protein extraction kit (Beyotime) according to the manufacturer’s instructions. Briefly, HCC cells were collected by cell scrapers, followed by three washes with cold PBS. Then, 1 mL of membrane protein extraction reagent A with PMSF was added. Cells were placed in an ice bath for 10 to 15 min. Then, cell lysates were centrifuged (700 × *g*) at 4 °C for 10 min, followed centrifugation (14,000 × *g*) for 30 min at 4 °C. The supernatant (cytoplasmic protein) was discarded to obtain membrane fraction-enriched lysates. Afterwards, 200 μL of membrane protein extraction reagent B was added, and cell lysates were resuspended and forcefully vortexed at high speed for 5 s, followed by incubation in an ice bath for 5 to 10 min. The vortexing and ice bath incubation steps were then repeated twice. The lysates were centrifuged (14,000 × *g*) at 4 °C for 5 min. The membrane proteins were enriched in the supernatants.

### Co-immunoprecipitation (Co-IP) assays

Co-IP assays were performed to determine the interactions between proteins according to a previous report with several modifications [22]. Briefly, the indicated cells were lysed in NP-40 lysis buffer containing 50 mM Tris–HCl (pH 7.4), 150 mM NaCl, and 1% NP-40, which was supplemented with protease inhibitor cocktail reagent (Beyotime). IP of protein complexes from cell lysates was performed using the following specific antibodies: ASAP2 (Santa Cruz, 1:25); CIN85 (Santa Cruz, 1:25); HA (CST, 1:50); Flag (CST, 1:50). Antibody-target complex was subsequently immobilized with Sepharose G beads (GE Life Science) and eluted under reducing-denaturing conditions in SDS lysis buffer and denatured at 95 °C for 5 min. Then, proteins were detected by western blot assays with specific antibodies as described above.

### Bioinformatics analysis

Gene Expression Profiling Interactive Analysis (GEPIA, http://gepia.cancer-pku.cn/), which sources data from The Cancer Genome Atlas (TCGA, https://tcga-data.nci.nih.gov/tcga/) and the Genotype-Tissue Expression project (GTEx, https://www.gtexportal.org/home/index.html) [23] were used to evaluate differential ASAP2 expression patterns in HCC patients with different disease stages and the association between ASAP2 and indicated genes. The Encyclopedia of RNA Interactomes (ENCORI) database was used to compare ASAP2 expression between HCC and normal liver samples [24]. The prognostic values of ASAP2 and CIN85 in TCGA dataset were evaluated by Kaplan–Meier (KM) Plotter (https://kmplot.com) [25]. Expression profiling data were extracted from TCGA Liver hepatocellular carcinoma (LIHC) dataset (https://portal.gdc.cancer.gov). HCC patients were stratified by their ASAP2 expression levels according to quartile range (0–25% as low; 75–100% as high) and differentially expressed genes (DEGs) were determined by comparing expression profiling results between the ASAP2-high and ASAP2-low groups using DESeq2 [1.36.0] and edgeR [3.38.2]. Gene set enrichment analysis (GSEA) was performed for DEGs using clusterProfiler [4.4.4]. Specifically, the gene set used for GSEA was based on the MSigDB database (https://www.gsea-msigdb.org/gsea/msigdb/collections.jsp). The STRING database (https://cn.string-db.org) was used for conducting protein–protein interaction (PPI) analysis, and WikiPathway analysis of PPI networks was performed by the Analysis Tab of the website. Gene expression omnibus (GEO) datasets including GSE62232, GSE36376, GSE112790, GSE121248, GSE6764, GSE19665, GSE39791, GSE36411 and GSE40367 were used for identifying differential expression of genes. BioGRID (https://thebiogrid.org) and HIPPIE (http://cbdm-01.zdv.uni-mainz.de/~mschaefer/hippie/index.php) datasets were used for identifying potential interactor of ASAP2.

### Statistical analysis

Statistical analyses were performed using SPSS v21.0 for Windows (IBM, Armonk, NY, USA). Quantification values are presented as mean ± standard deviation (SD). Continuous data were analyzed by one-way ANOVA, and data that were normally distributed were analyzed using the two-tailed Student's *t*-test. Categorical variables were analyzed by the chi-square tests. Relationships between clinicopathological characteristics were investigated by the chi-square test or Fisher's exact test. Kaplan–Meier curve analysis, the log-rank test, and Cox regression analysis were applied for survival analysis. A *P*-value < 0.05 was considered statistically significant.

## Results

### ASAP2 is upregulated in HCC, partially resulting from genomic variation

**Fig. 1:**
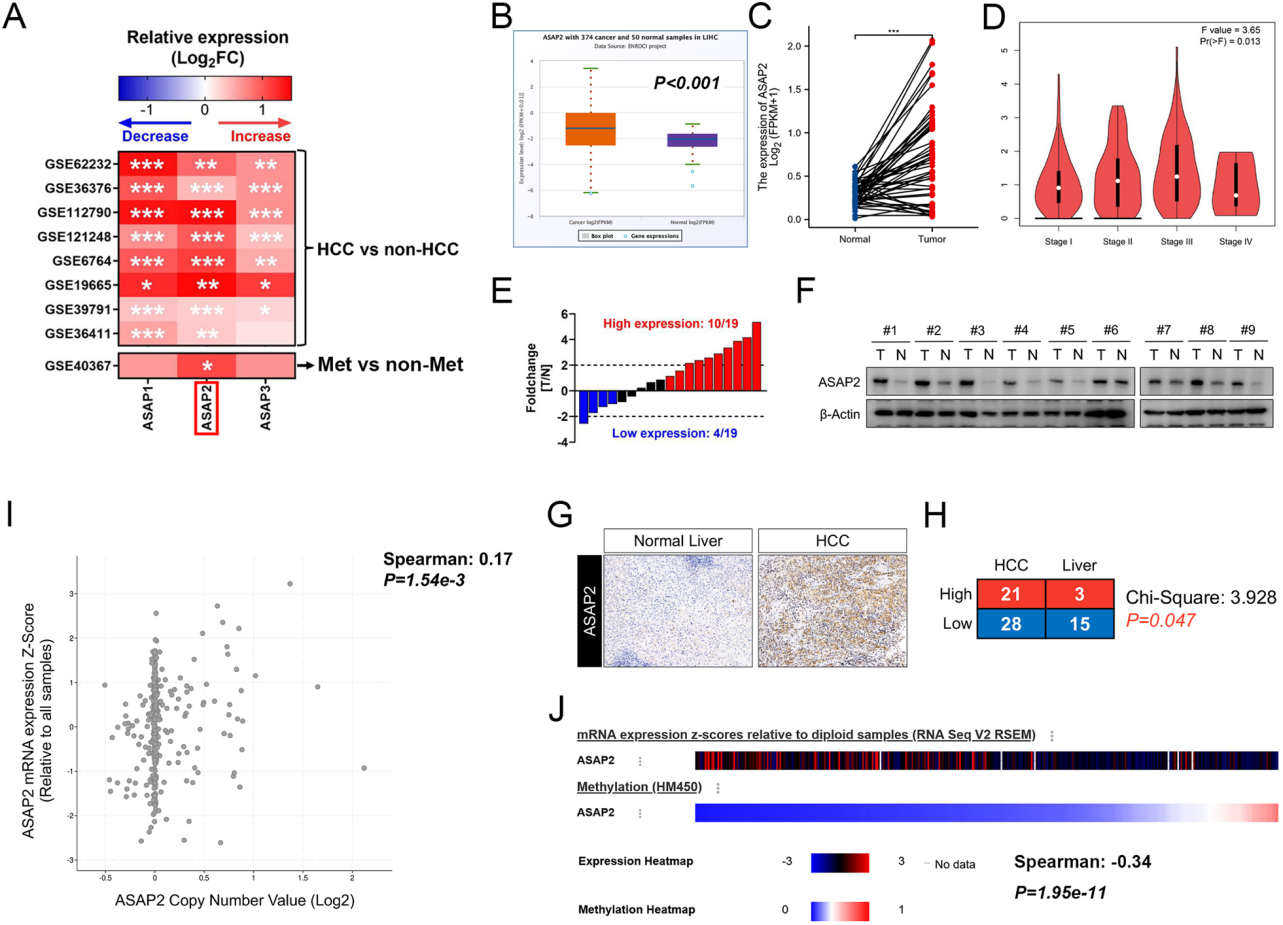
ASAP2 is upregulated in hepatocellular carcinoma (HCC). **A** Heatmap for expression patterns of ASAP1, ASAP2, and ASAP3 in the indicated gene expression omnibus (GEO) datasets; “*” indicates *0.01* < *P* < *0.05,* “**” indicates *0.001* < *P* < *0.01,* “***” indicates *P* < *0.001*. **B** Comparison of ASAP2 expression levels between normal liver and HCC tissues in The Cancer Genome Atlas (TCGA) dataset according to the Starbase 3.0 dataset. **C** Comparison of ASAP2 expression levels between paired normal liver and HCC tissues in TCGA dataset. **D** ASAP2 expression levels in HCC patients with different clinical stages in TCGA dataset according to the Gene Expression Profiling Interactive Analysis (GEPIA) dataset. **E** qRT-PCR results of ASAP2 mRNA expression levels between paired normal liver and HCC tissues collected from Fudan University Shanghai Cancer Center (FUSCC). **F** Western blot results of ASAP2 protein expression levels between paired normal liver and HCC tissues collected from FUSCC. **G** Representative immunohistochemistry staining images for ASAP2 in normal liver and HCC tissues collected from FUSCC. **H** Distribution of ASAP2 expression states in normal liver and HCC tissues collected from FUSCC. **I** Correlation between DNA copy number and mRNA expression levels of ASAP2 in HCC in TCGA dataset according to the cbioportal dataset. **J** Association between mRNA expression levels and DNA methylation pattern of ASAP2 in HCC according to TCGA dataset

First, we observed the expression patterns of ASAP2 and the other two major ASAP proteins, ASAP1 and ASAP3, in normal liver and HCC tissues according to the gene expression omnibus (GEO) dataset. We found that ASAP1 and ASAP2 expression levels were greatly upregulated in all datasets enrolled. Next, we compared the expression levels of these three proteins in metastatic and non-metastatic primary HCC tissues, and the results showed that only ASAP2 expression was significantly upregulated in metastatic HCC tissues from the GSE40367 dataset (Fig. 1A). Interestingly, the GTEx, HPA, and FANTOM5 datasets all demonstrated low expression of ASAP2 in normal liver tissues (Additional file 1: Fig. S1A–C), suggesting a potential oncogenic role of ASAP2, especially in promoting disease progression and metastasis. Moreover, ASAP2 was significantly elevated in HCC tissues (Fig. 1B) and greatly increased in HCC tissues relative to their corresponding, paired normal liver tissues in TCGA dataset (Fig. 1C). Notably, ASAP2 expression gradually increased with progressing tumor stage (*P* = 0.013, Fig. 1D). Further analysis revealed that ASAP2 also exhibited higher expression levels in other types of malignancies, such as cholangiocarcinoma, head and neck squamous cell carcinoma, and lung squamous cell carcinoma (Additional file 1: Fig. S1D).

To validate these results from public datasets, we collected HCC tissues and paired adjacent normal liver tissue (ANLT) from patients undergoing curative resection in FUSCC, then performed qRT-PCR and western blot assays. The results demonstrated that ASAP2 expression was greatly enhanced in HCC tissues (Fig. 1E, F). IHC images of ASAP2 protein expression in HCC tissues and ANLTs are shown in Fig. 1G. IHC assays suggested that HCC tissues had higher ASAP2 protein expression (*P* = 0.047, Fig. 1H). Genomic alteration of ASAP2 was further investigated, and we observed a significantly positive correlation between ASAP2 mRNA expression levels and ASAP2 DNA copy number in the TCGA dataset (*P* < 0.001, Fig. 1I). On the contrary, methylation levels were negatively associated with ASAP2 mRNA expression levels (*P* < 0.001, Fig. 1J). Collectively, these investigations indicate that ASAP2 is pathologically elevated in HCC.

### Increased ASAP2 predicts poor prognosis in HCC

**Fig. 2:**
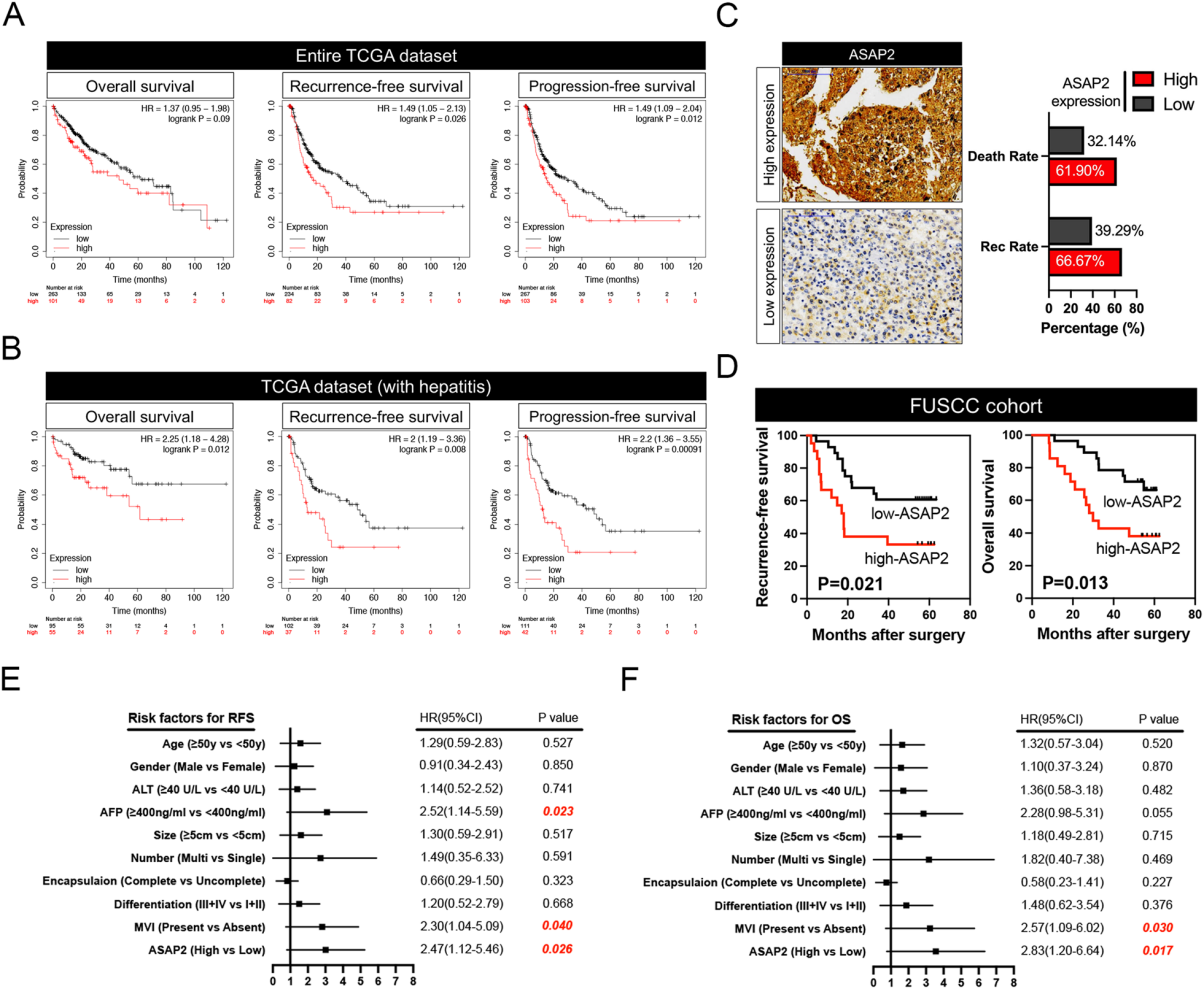
Elevated ASAP2 expression levels predict poor prognosis in hepatocellular carcinoma (HCC). **A** Prognostic value of ASAP2 in predicting overall survival (OS), recurrence-free survival (RFS), and progression-free survival (PFS) in the entire The Cancer Genome Atlas (TCGA) Liver hepatocellular carcinoma (LIHC) cohort. **B** Prognostic value of ASAP2 in predicting OS, RFS, and PFS in patients with hepatitis in TCGA LIHC cohort. **C** Typical images of ASAP2 expression in HCC tissues collected from Fudan University Shanghai Cancer Center (FUSCC, left), and death and recurrence rates of HCC patients with distinct ASAP2 states (right). **D** Kaplan–Meier curve analysis of ASAP2 expression for predicting RFS (left) and OS (right) in patients from FUSCC. **E**, **F** Univariate Cox regression analysis of ASAP2 and other clinicopathological parameters for predicting RFS (left) and OS (right) in patients from FUSCC

We further investigated the prognostic significance of ASAP2 in HCC. First, we stratified the HCC patients in TCGA dataset according to their ASAP2 expression levels and performed GSEA (stratification details are outlined in the Materials and Methods section). The results revealed that the WOO_LIVER_CANCER_RECURRENCE_UP signature was positively enriched, while the survival-related signature was negatively associated with ASAP2-high HCC (Additional file 1: Fig. S2A). Kaplan–Meier analysis suggested that high ASAP2 levels indicate significant shorter RFS and progression-free survival (PFS) in the entire TCGA LIHC cohort (Fig. 2A). Similarly, ASAP2 had prognostic value for predicting RFS, PFS, and OS in HCC patients with hepatitis (Fig. 2B) according to TCGA dataset. We next explored the prognostic significance of ASAP2 in the FUSCC cohort we established. As shown in Fig. 2C, patients with high ASAP2 levels were prone to dismal outcomes. Kaplan–Meier analysis demonstrated that the high ASAP2 group had significantly shorter RFS and OS (Fig. 2D). However, ASAP2 did not exhibit a significant correlation with other clinicopathological parameters (Additional file 1: Fig. S2B). Univariate Cox regression analysis revealed that ASAP2 is a significant indicator for RFS in the FUSCC cohort, in addition to α-fetoprotein (AFP) and microvascular invasion (MVI, Fig. 2E). Importantly, high ASAP2 expression was also identified as a novel indicator for OS by univariate Cox regression (Fig. 2F). Intriguingly, high ASAP2 levels could also predict poor prognosis across several types of malignancies, including cervical, lung, pancreatic, and uterine cancers (Additional file 1: Fig. S3A–D), suggesting that ASAP2 is a common contributor to cancer progression. Collectively, our data imply that upregulation of ASAP2 is a potential promising prognostic predictor in HCC.

### ASAP2 promotes cell proliferation and inhibits apoptosis in HCC cells

**Fig. 3:**
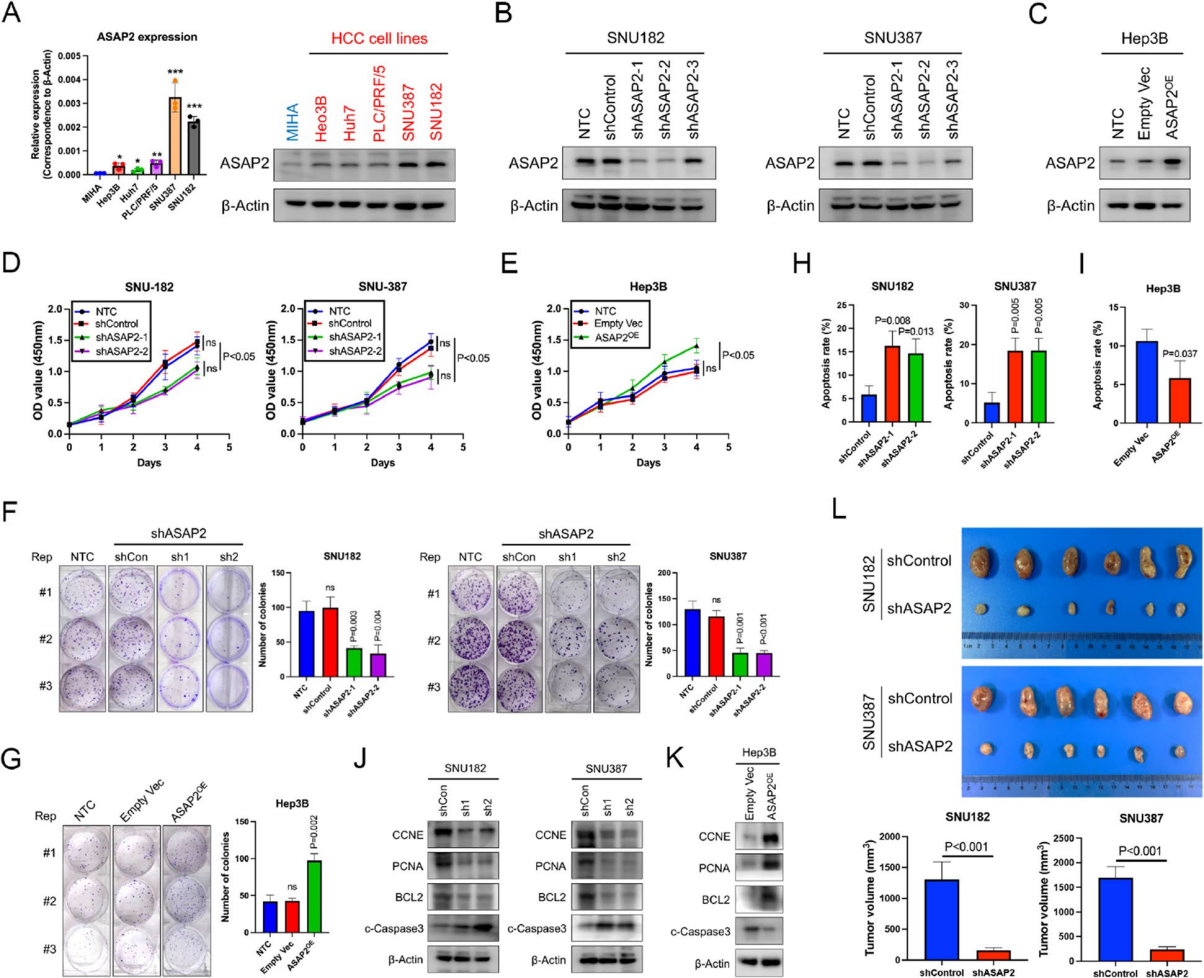
ASAP2 promotes hepatocellular carcinoma (HCC) growth. **A** ASAP2 expression states in normal and HCC cells as determined by qRT-PCR and western blot (WB) assays. **B** ASAP2 knockdown efficiencies in SNU182 (left) and SNU387 (right) cells were assessed by WB assays. **C** ASAP2 overexpression efficiency in Hep3B cells was evaluated by WB assays. **D** The proliferation rates of SNU182 (left) and SNU387 (right) cells with ASAP2 knockdown cultured in DMEM supplemented with 10% fetal bovine serum (FBS) were evaluated by cell counting kit-8 (CCK8) assays. **E** Proliferation rates of Hep3B cells with ASAP2 overexpression cultured in DMEM supplemented with 10% FBS were evaluated by CCK8 assays. **F** Proliferation rates of SNU182 (left) and SNU387 (right) cells with ASAP2 knockdown were evaluated by colony formation assays; Cells were cultured in DMEM supplemented with 10% FBS for 14 days and stained with crystal violet. **G** Proliferation rates of Hep3B cells with ASAP2 overexpression were evaluated by colony formation assays; Cells were cultured in DMEM supplemented with 10% FBS for 14 days and stained with crystal violet. **H** Apoptosis rates in SNU182 (left) and SNU387 (right) cells after ASAP2 knockdown were evaluated by flow cytometry analysis. **I** Apoptosis rates in Hep3B cells after ASAP2 overexpression were evaluated by flow cytometry analysis. **J** Protein expression levels of the indicated markers after ASAP2 knockdown in SNU182 (left) and SNU387 (right) cells were assessed by WB assays. **K** Protein expression levels of the indicated markers after ASAP2 overexpression in Hep3B cells were assessed by WB assays. **L** Mice were injected with shControl-SNU182/SNU387 or ASAP2-knockdown SNU182/SNU387 cells to generate subcutaneous mice models. Recipient mice were sacrificed 4 weeks after transplantation; Tumor tissues are shown in the upper panel (n = 6 per group) and the corresponding tumor volumes are shown in the lower panel. “ns” indicates no significance

Western blot and qRT-PCR assays indicated ASAP2 expression levels were increased in HCC cells compared with those in a normal liver cell line, MIHA (Fig. 3A). Because ASAP2 expression levels were highest in SNU387 and SNU182 cells, these two cell lines were selected for further knockdown experiments. Hep3B cells showed the lowest ASAP2 expression levels of the cell lines tested and were therefore chosen for ASAP2 overexpression experiments. The knockdown and overexpression efficiencies were validated by western blot assays (Fig. 3B, C). The CCK-8 assays demonstrated that knockdown of ASAP2 significantly reduced cell proliferation, while ASAP2 overexpression achieved the opposite effect (Fig. 3D, E). Similarly, ASAP2 downregulation reduced HCC cell colony numbers (Fig. 3F), and ectopic ASAP2 expression markedly promoted cell proliferation ability (Fig. 3G). Moreover, knocking down ASAP2 significantly increased apoptosis rates in HCC cells (Fig. 3H), whereas ASAP2 overexpression greatly reduced apoptosis (Fig. 3I). Further western blot assays indicated decreased protein expression levels of proliferation and anti-apoptosis-related markers, such as CCNE, PCNA, and BCL2, but upregulation of cleaved-Caspase 3, a typical marker for apoptosis, in ASAP2-knockdown cells (Fig. 3J). However, protein expression patterns of these markers were completely reversed in ASAP2-overexpression cells (Fig. 3K). Consistent with these in vitro experiment results, GSEA results indicated an enhanced proliferation capacity and accelerated cell cycle signatures in ASAP2-high HCC according to TCGA dataset (Additional file 1: Fig. S4A, B), while apoptosis-related signatures were positively enriched in the ASAP2-low group (Additional file 1: Fig. S4C). Accordingly, positive correlations between ASAP2 and proliferation/cell cycle-related markers (MKI67, PCNA, MYC, CCND, CCNE, CDK2, CDK4 and CDK6) or anti-apoptosis (BCL2, XIAP, BIRC3, and BIRC2) were observed (Additional file 1: Fig. S4D, E). Finally, subcutaneous tumor transplantation experiments were performed, and the results demonstrated that silencing ASAP2 could significantly impair tumor growth in vivo, as evidenced by reduced tumor volumes (Fig. 3L). Thus, these data strongly imply that ASAP2 is a crucial regulator for sustaining HCC growth.

### ASAP2 is essential for the migration and invasion capacities of HCC cells

**Fig. 4:**
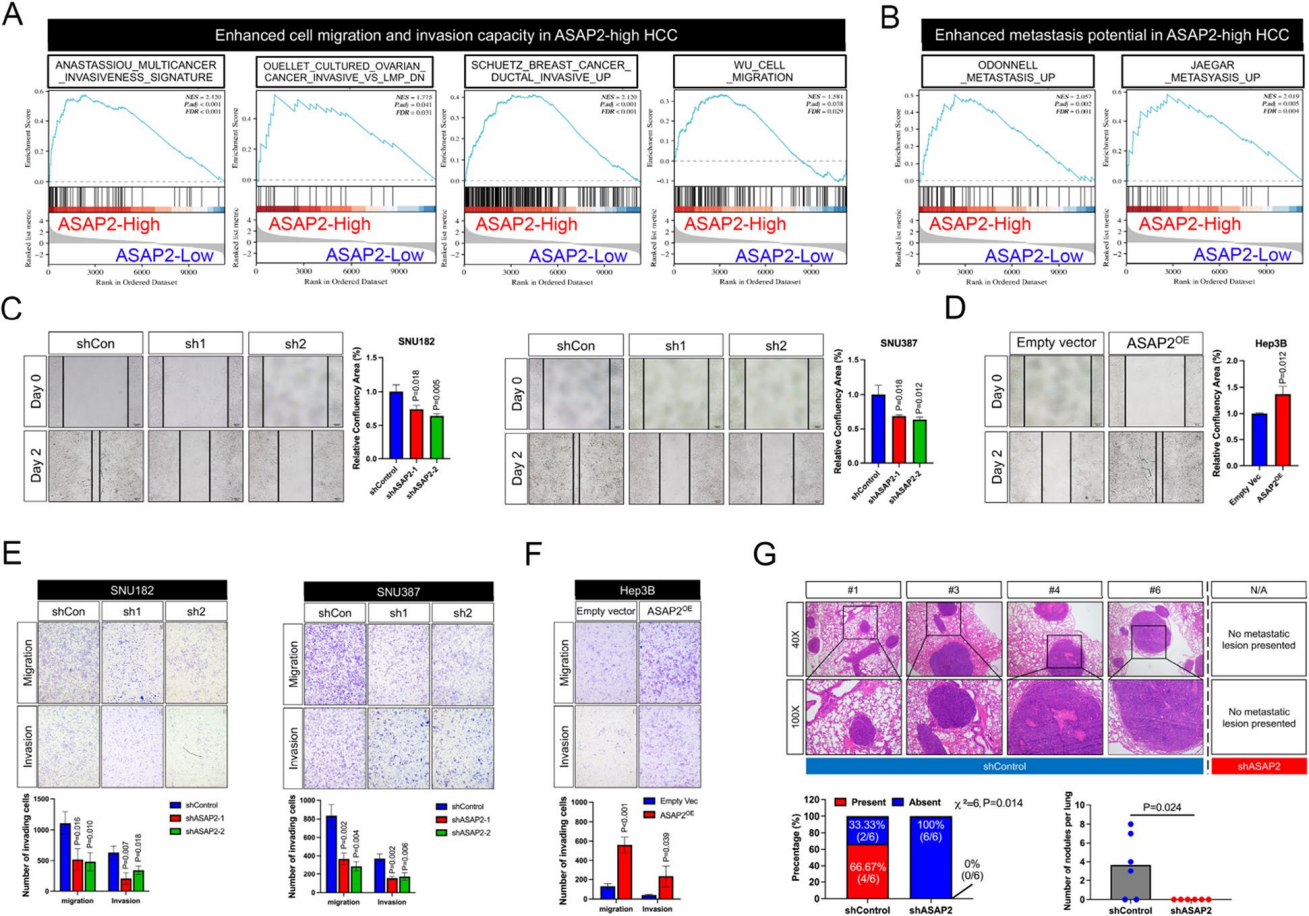
ASAP2 facilitates hepatocellular carcinoma (HCC) migration and invasion. **A** Associations between migration/invasion-related molecular signatures and ASAP2 expression in The Cancer Genome Atlas (TCGA) Liver hepatocellular carcinoma (LIHA) cohort according to gene set enrichment analysis (GSEA). **B** Metastasis-related molecular signatures were enriched in ASAP2-high HCC samples according to TCGA dataset. **C** Effects of ASAP2 knockdown on the migration rates of SNU182 (left) and SNU387 (right) cells were assessed by wound healing experiments. **D** Effects of ASAP2 overexpression on the migration rates of Hep3B cells were assessed by wound healing experiments. **E** Effects of ASAP2 knockdown on the invasion capacity of SNU182 (left) and SNU387 (right) cells were assessed by Transwell experiments. **F** Effects of ASAP2 overexpression on the invasion capacity of Hep3B cells were assessed by Transwell experiments. **G** shControl or shASAP2 SNU182 cells were injected into recipient mice through the tail vein to establish lung metastasis models; Representative images of the metastases are illustrated in the left panel and the incidence of lung metastases are shown in the right panel

To investigate the role of ASAP2 in HCC metastasis, we first conducted GSEA on TCGA dataset. Cell migration and invasion-related signatures were significantly enriched in ASAP2-high HCC (Fig. 4A). Moreover, ASAP2-high HCC also had a molecular signature that correlated with an increased metastatic potential (Fig. 4B). These findings suggest that ASAP2 can promote metastasis in HCC. Next, wound healing assays showed that ASAP2 knockdown greatly impaired cell migration rates (Fig. 4C), while ASAP2 overexpression exerted the opposite effect in Hep3B cells (Fig. 4D). Similarly, Transwell assays demonstrated that HCC cell migration and invasion capacities were significantly reduced when ASAP2 expression was knocked down (Fig. 4E). Increased mobility and invasion were supported by ASAP2 overexpression in Hep3B cells according to Transwell assays (Fig. 4F). To verify the critical role of ASAP2 in HCC metastasis in vivo, tail vein injections were applied to generate pulmonary metastasis mice models. ASAP2 knockdown greatly inhibited the incidence of lung metastasis (shControl 4/6 vs. shASAP2 0/6, P = 0.014). Also, shControl SNU182 cells generated significantly higher metastatic nodules in lung (P = 0.024, Fig. 4G). Collectively, these data indicate that ASAP2 has a vital role in promoting HCC migration and invasion both in vitro and in vivo.

### ASAP2 triggers EMT in HCC

**Fig. 5:**
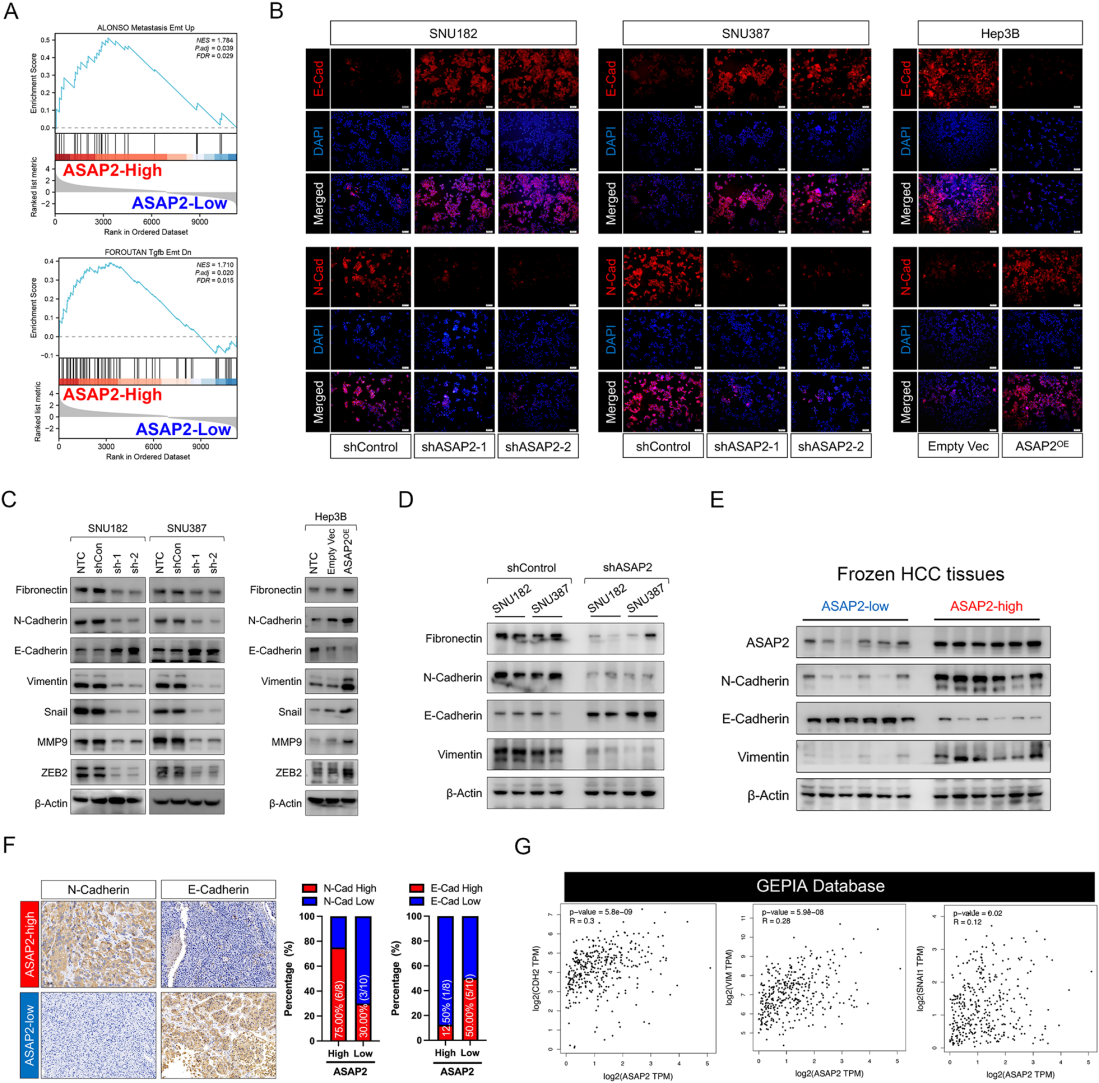
ASAP2 triggers the epithelial-mesenchymal transition (EMT) in hepatocellular carcinoma (HCC). **A** Gene set enrichment analysis (GSEA) results indicated that EMT-related molecular signatures were significantly enriched in ASAP2-high HCC samples according to The Cancer Genome Atlas (TCGA) dataset. **B** Expression levels of N-Cadherin and E-Cadherin in ASAP2-knockdown SNU182 cells, ASAP2-knockdown SNU387 cells, ASAP2-overexpression Hep3B cells and their corresponding control cells were evaluated by immunofluorescence staining. **C** Effects of ASAP2 knockdown (left) or ASAP2 overexpression (right) on the protein expression levels of EMT-related markers in HCC cells were determined by western blot (WB) assays. **D** Protein expression levels of EMT-related markers in HCC tissues derived from mice models generated by transplanting shControl or shASAP2-SNU182/SNU387 cells were assessed by WB assays. **E** Protein expression levels of EMT-related markers in frozen HCC samples with distinct ASAP2 states were determined by WB assays. **F** Expression patterns of N-Cadherin and E-Cadherin in patients with distinct ASAP2 states in clinical HCC samples were determined by immunohistochemistry staining. **G** Associations between ASAP2 and EMT-related markers such as N-Cadherin, Vimentin, and Snail1 according to TCGA Liver hepatocellular carcinoma (LIHC) cohort and the Gene Expression Profiling Interactive Analysis (GEPIA) dataset

EMT was identified as a driving force of HCC recurrence [26]. Therefore, we conducted GSEA for ASAP2 with TCGA dataset to investigate the potential correlation between ASAP2 and EMT in HCC. As expected, EMT-related signatures were significantly enriched in ASAP2-high HCC (Fig. 5A). Immunofluorescence staining suggested that the protein levels of N-Cadherin, a typical marker for a mesenchymal-like phenotype, were greatly decreased when ASAP2 expression was knocked down. However, protein expression levels of E-Cadherin, a marker of an epithelial-like phenotype, were greatly enhanced (Fig. 5B). Notably, ectopic expression of ASAP2 resulted in the opposite effects on E-Cadherin and N-Cadherin protein expression in Hep3B cells. Western blot and qRT-PCR assays indicated that ASAP2 knockdown resulted in reduced expression levels of mesenchymal-related molecules, including Fibronectin, Vimentin, α-SMA, Snail, Zeb2, and MMP9, but significantly upregulated E-Cadherin expression levels (Fig. 5C left panel, Additional file 1: Fig. S5A). ASAP2 overexpression promoted mesenchymal-related molecule levels, but downregulated E-Cadherin expression levels in Hep3B cells (Fig. 5C right panel, Additional file 1: Fig. S5B). Next, we examined the expression levels of EMT-related molecules in HCC tissues derived from the subcutaneous transplanted mice models. Both qRT-PCR and western blot assays demonstrated that knocking down ASAP2 expression resulted in reduced Fibronectin, N-Cadherin, and Vimentin expression levels, but enhanced E-Cadherin levels (Fig. 5D and Additional file 1: Fig. S5C). Additionally, western blot assays confirmed that ASAP-high HCC clinical samples had increased N-Cadherin and Vimentin expression levels, but decreased E-Cadherin levels (Fig. 5E). IHC assays also indicated that ASAP2-high HCC tissues tended to have higher N-Cadherin protein expression levels, but lower E-Cadherin protein expression (Fig. 5F). Moreover, ASAP2 exhibited a significantly positive correlation with mesenchymal-related markers such as N-Cadherin, Vimentin, Fibronectin, Zeb2, and MMP3 in TCGA dataset (Fig. 5G and Additional file 1: Fig. S5D). Taken together, these findings emphasize the role of ASAP2 in triggering EMT in HCC, as well as the importance of maintaining a mesenchymal phenotype to facilitate HCC invasion.

### ASAP2 is required for an HGF/c-MET signaling-induced malignant phenotype in HCC

**Fig. 6:**
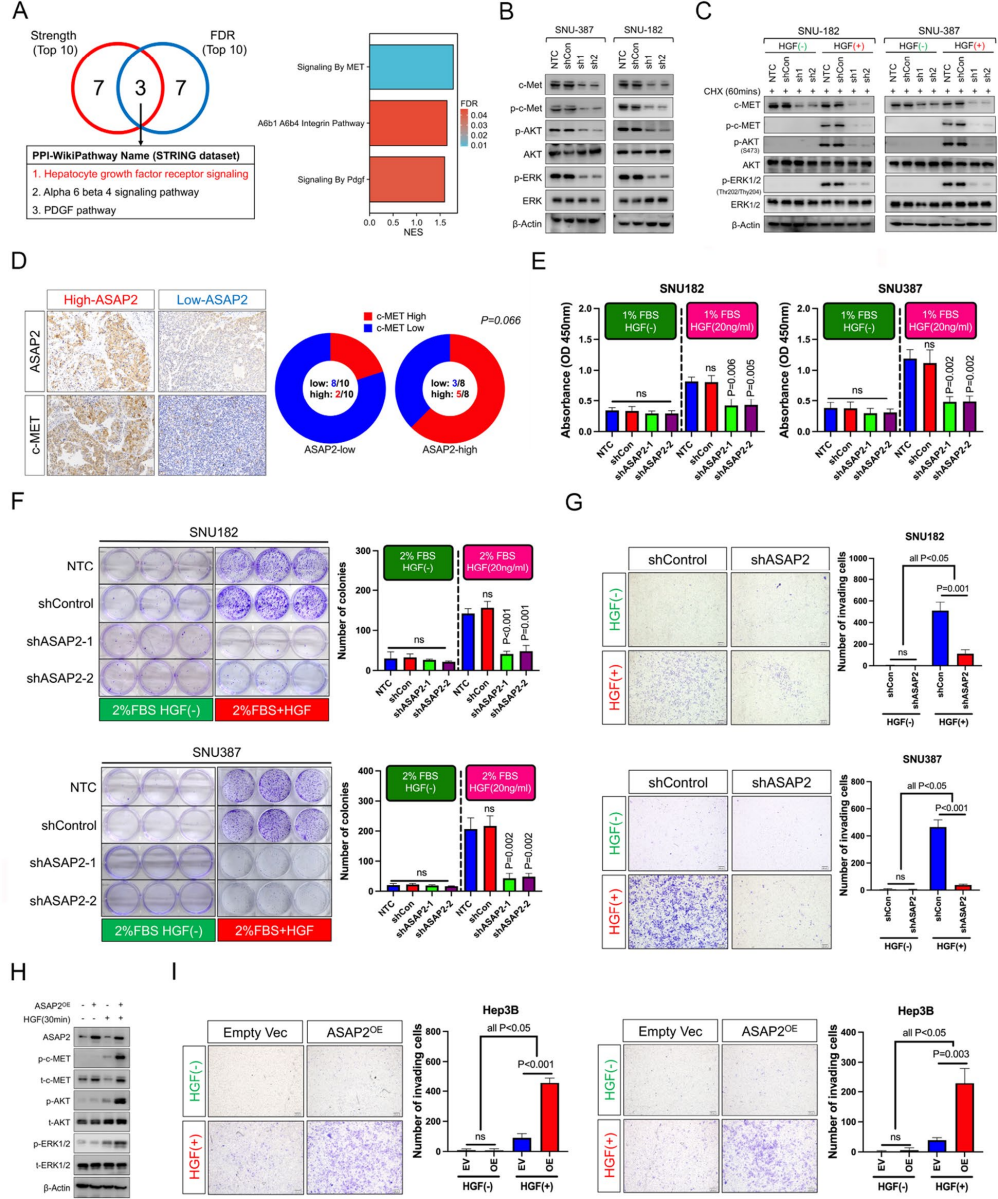
ASAP2 is essential for HGF/c-MET signaling-induced malignant phenotypes in hepatocellular carcinoma (HCC). **A** Venn diagram showing the overlapping WikiPathways from the top 10 signaling pathways ranked by protein–protein interaction (PPI) strength and false discovery rate (FDR) value (Left); Histogram showing normalized enrichment score (NES) for the above mentioned three overlapping signaling pathways and the color indicated FDR value (right). **B** Effects of ASAP2 knockdown on the levels of total c-MET, p-c-MET, p-AKT, and p-ERK1/2 in SNU387 (left) and SNU182 (right) cells cultured in DMEM supplemented with 10% fetal bovine serum (FBS) were determined by western blot (WB) assays. **C** shControl or ASAP2-knockdown SNU182 (left)/SNU387 (right) cells were serum starved for 12 h; Afterwards, the culture medium was replaced with serum-free DMEM with or without hepatocyte growth factor (HGF, 20 ng/mL) plus Cycloheximide (CHX, 50 μg/mL), and cells were cultured for 60 min; Total protein was extracted, followed by WB analysis to determine the effects of ASAP2 knockdown on the levels of total c-MET, p–c-MET, p-AKT, and p-ERK1/2 in HCC cells upon HGF stimulation. **D** Association between ASAP2 and c-MET expression levels in clinical HCC samples. **E** shControl or ASAP2-knockdown SNU182 (left)/SNU387 (right) cells were serum starved for 12 h; Afterwards, the culture medium was replaced with DMEM containing 1% FBS with or without HGF (20 ng/mL), and cells were cultured for 3 days; At day 3, Cell Counting Kit-8 (CCK8) assays were conducted to evaluate the effects of ASAP2 knockdown on HCC cell proliferation rates following HGF stimulation. **F** shControl or ASAP2-knockdown SNU182 (upper)/SNU387(lower) cells were seeded into 6-well plates at a density of 1500 cells per well; After culturing in DMEM containing 10% FBS for 24 h to allow complete cell attachment to the plate, the medium was replaced with DMEM containing 2% FBS with or without HGF (20 ng/mL). The medium was replaced twice a week; At Day 14, the cells were stained with crystal violet to assess the influence of ASAP2 knockdown on the colony-formation capacity of HCC cells upon HGF stimulation. **G** shControl or ASAP2-konckdown SNU182 (upper)/SNU387 (lower) cells were seeded into Transwell chamber inserts (8 μm pore size, upper chamber) at a density of 5 × 10^4^ cells per well; The upper chamber was supplemented with DMEM containing 1% FBS, while the lower chamber was supplemented with DMEM containing 1% FBS with or without HGF (20 ng/mL); Cells were cultured for 24 h to allow invasion; Afterwards, the upper chambers were collected and the invading cells were quantified with crystal violet staining to evaluate the influence of ASAP2 knockdown on invasion rates of HCC cells upon HGF stimulation. **H** Control and ASAP2-overexpression Hep3B cells were serum starved for 12 h, then the culture medium was replaced with serum-free DMEM with or without HGF (20 ng/mL); Cells were treated for 30 min and protein was extracted, then WB assays were conducted to evaluate the influence of ASAP2 overexpression on the levels of total c-MET, p–c-MET, p-AKT, and p-ERK1/2 in HCC cells upon HGF stimulation. **I** Control and ASAP2-overexpression Hep3B cells were seeded into Transwell chamber inserts (8 μm pore size, upper chamber) at a density of 5 × 10^4^ cells per well; The upper chamber was supplemented with DMEM containing 1% FBS, while the lower chamber was supplemented with DMEM containing 1% FBS with or without HGF (20 ng/mL); Cells were cultured for 24 h to allow migration or invasion; Afterwards, the upper chambers were collected and the invading cells were quantified with crystal violet staining to evaluate the influence of ASAP2 overexpression on migration (left) and invasion (right) rates of HCC cells upon HGF stimulation

We next sought to investigate the downstream signaling of ASAP2 in HCC. Because ASAP2 mainly exerts its function via interactions with other proteins, we first explored the ASAP2 protein–protein interaction (PPI) network according to the STRING dataset. Venn diagram analysis identified three overlapping WikiPathways with greater strength and more significant FDR, suggesting that these pathways were likely downstream of ASAP2 (Fig. 6A left). Interestingly, HGF/c-MET signaling, a previously reported major driver of EMT in HCC [27], was identified. Further GSEA results showed that HGF/c-MET signaling was significantly enriched in the ASAP2-high group, and exhibited a higher enrichment score than the A6B4 integrin and PDGF pathways, another two signaling identified by PPI analysis (Fig. 6A right). Moreover, signatures of several downstream targets of c-MET signaling, such as MET Activates PTK2 Signaling, MET Promotes Cell Motility, and PI3K-AKT Signaling, were also enriched in the ASAP2-high HCC according to GSEA (Additional file 1: Fig. S6A). Therefore, we mainly focused on HGF/c-MET signaling.

Western blot results showed strong downregulation of phosphorylated c-MET, AKT, and ERK1/2 after ASAP2 knockdown (Fig. 6B). Notably, total c-MET levels were also decreased, suggesting that ASAP2 could also affect total c-MET protein. Importantly, ASAP2 knockdown weakened the responsiveness of HCC cells towards HGF stimulation, as evidenced by greatly reduced levels of phosphorylated c-MET, AKT, and ERK1/2 in ASAP2-knockdown cells after HGF treatment (Fig. 6C). Clinically, ASAP2-high HCC tissues also tended to exhibit higher c-MET expression levels (Fig. 6D). Functionally, ASAP2 knockdown could significantly inhibit HCC cell proliferation upon HGF stimulation according to both CCK8 (Fig. 6E) and colony formation assays (Fig. 6F), while no significant proliferation difference was observed before and after ASAP2 knockdown when HGF was absent. The enhanced migration and invasion capacities following HGF stimulation could also be greatly alleviated by ASAP2 knockdown (Fig. 6G, Additional file 1: Fig. S6B). More importantly, the degree of c-MET signaling activation after HGF stimulation was higher in ASAP2-overexpression Hep3B cells compared with that in the control cells, as evidenced by increased phosphorylated c-MET, AKT, and ERK1/2 levels (Fig. 6H). Moreover, the migration, invasion (Fig. 6I), and proliferation (Additional file 1: Fig. S6C) capacities were significantly enhanced in ASAP2-overexpression Hep3B cells upon HGF stimulation compared with those in control cells. However, ASAP2 overexpression had limited effects on HCC cells without HGF stimulation. Together, our data indicated ASAP2 was essential for the HGF/c-MET signaling activation.

### ASAP2 interacts with CIN85 to disrupt the CIN85-c-EMT association and prevent activated c-MET degradation

**Fig. 7:**
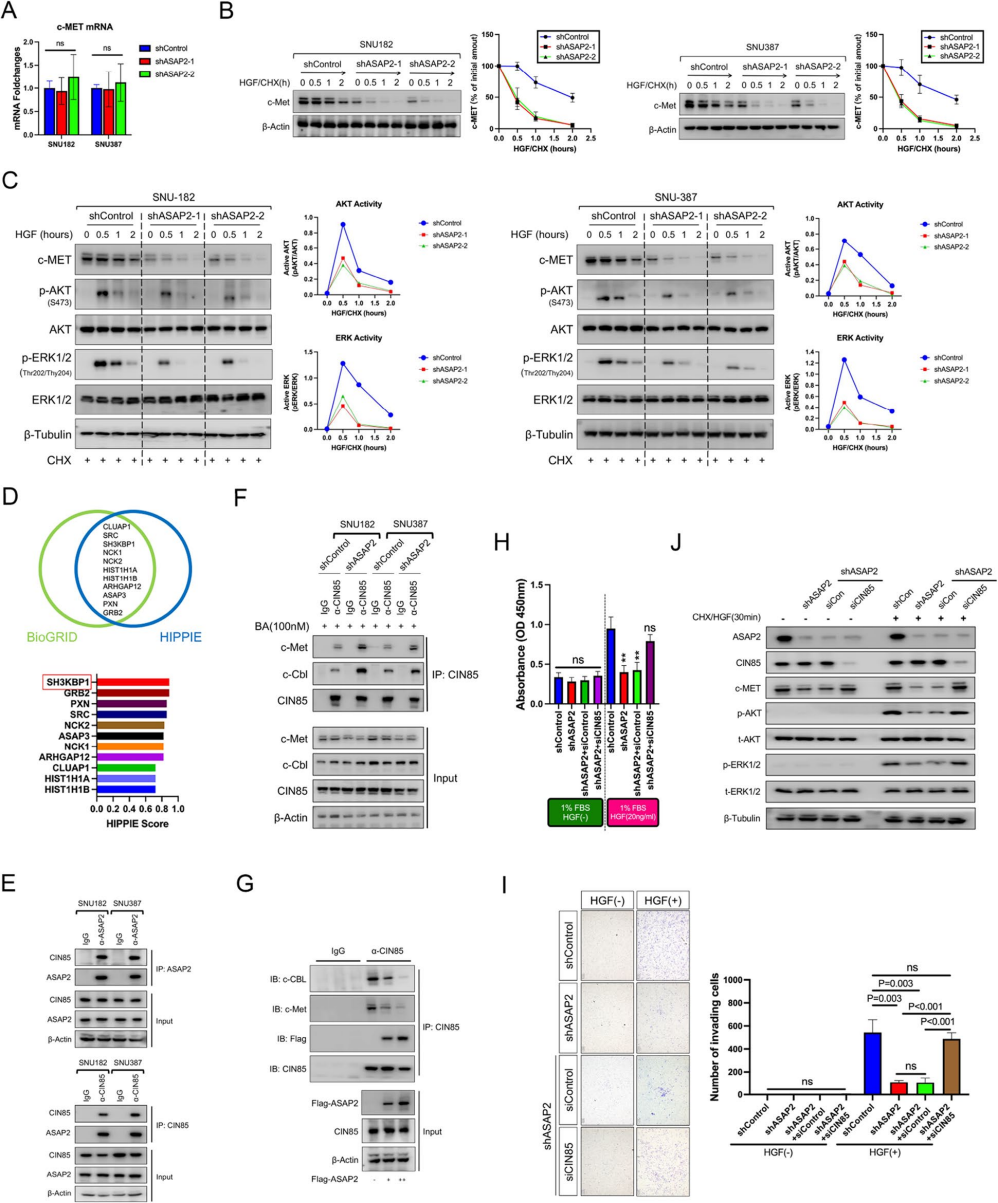
ASAP2 binds to CIN85 to interrupt the CIN85-c-MET interaction and subsequent c-MET degradation. **A** Alteration of mRNA expression levels of c-MET from ASAP2 knockdown according to qRT-PCR. **B** shControl and ASAP2-knockdown SNU182 (left)/SNU387 (right) cells were serum starved for 12 h; Afterwards, the culture medium was replaced with serum-free DMEM with or without hepatocyte growth factor (HGF, 20 ng/mL) plus Cycloheximide (CHX, 50 μg/mL). Protein was extracted at the indicated time point, followed by western blot (WB) assays to evaluate the influence of ASAP2 knockdown on the half-life of c-MET protein. **C** shControl and ASAP2-knockdown SNU182 (left)/SNU387 (right) cells were serum starved for 12 h; Afterwards, the culture medium was replaced with serum-free DMEM with or without HGF (20 ng/mL) plus CHX (50 μg/mL); Protein was extracted at the indicated time point, followed by WB assays to evaluate the influence of ASAP2 knockdown on the p-AKT and p-ERK1/2 levels in HCC cells upon HGF stimulation. **D** Venn diagram demonstrating the overlapping ASAP2-interactors between the BioGRID and HIPPIE databases (upper), and HIPPIE scores for overlapping proteins (lower). **E** ASAP2 was pulled down, followed by WB assays to demonstrate the interaction between ASAP2 and CIN85 in HCC cells (upper); Then, CIN85 was pulled down, followed by WB assays to demonstrate the interaction between ASAP2 and CIN85 in HCC cells (lower). **F** The effects of ASAP2 on the interaction between CIN85 and c-MET in HCC cells were determined by immunoprecipitation (IP) experiments, followed by WB assays; HCC cells were treated with Bafilomycin A1 (BA, 100 nM) to prevent c-MET degradation as much as possible. **G** Increased abundance of exogenous ASAP2 expressed in Hep3B cells and its impacts on the interaction between endogenous c-MET and CIN85 were determined by IP followed by WB assays. **H** shControl SNU182 cells, ASAP2-knockdown SNU182 cells, ASAP2-knockdown SNU182 cells transfected with control siRNA, and ASAP2-knockdown SNU182 cells transfected with siCIN85 were serum starved for 12 h; Afterwards, the culture medium was replaced with DMEM containing 1% FBS with or without HGF (20 ng/mL) and the cells were cultured for 3 days; At day 3, Cell Counting Kit-8 (CCK8) assays were conducted to evaluate the proliferation rates. **I** shControl SNU182 cells, ASAP2-knockdown SNU182 cells, ASAP2-knockdown SNU182 cells transfected with control siRNA, and ASAP2-knockdown SNU182 cells transfected with siCIN85 were seeded into Transwell chamber inserts (8 μm pore size, upper chamber) at a density of 5 × 10^4^ cells per well; The upper chamber was supplemented with DMEM containing 1% FBS, while the lower chamber was supplemented with DMEM containing 1% FBS with or without HGF (20 ng/mL); Cells were cultured for 24 h to allow invasion; Afterwards, the upper chambers were collected and the invading cells were quantified with crystal violet staining to evaluate the invasion rates. **J** shControl SNU182 cells, ASAP2-knockdown SNU182 cells, ASAP2-knockdown SNU182 cells transfected with control siRNA, and ASAP2-knockdown SNU182 cells transfected with siCIN85 were serum starved for 12 h; Afterwards, the culture medium was replaced with serum-free DMEM with or without HGF (20 ng/mL) plus CHX (50 μg/mL), and the cells were cultured for 30 min; Total protein was extracted, followed by WB assays to determine the effects of ASAP2 knockdown on the levels of total c-MET, p–c-MET, p-AKT, and p-ERK1/2 of HCC cells upon HGF stimulation. “**” indicates *0.001* < *P* < *0.01*

Because qRT-PCR assays demonstrated that ASAP2 knockdown did not downregulate c-MET mRNA expression (Fig. 7A), we hypothesized that ASAP2 possibly regulates c-MET expression at the post-transcriptional level. To validate this, CHX chasing experiments were performed, which indicated significantly reduced c-MET protein stability after ASAP2 knockdown (Fig. 7B). Moreover, phosphorylated AKT and ERK1/2 levels were also attenuated after HGF stimulation when ASAP2 expression was knocked down (Fig. 7C). Co-IP experiments indicated greatly decreased interactions between c-MET and Gab1, a vital intracellular adaptor for transmitting active c-MET signals. Accordingly, phosphorylated Gab1 levels were also greatly reduced following ASAP2 knockdown (Additional file 1: Fig. S7 left). Contrarily, ASAP2 overexpression induced enhanced interactions between c-MET and Gab1 and promoted phosphorylation of Gab1 (Additional file 1: Fig. S7 right). These findings emphasize the necessity of ASAP2 in c-MET signal transduction. Previous studies have reported that membrane c-MET is rapidly internalized and degraded after being activated by HGF [22]. Thus, we separated membrane protein, then conducted western blot assays. We found that c-MET showed moderate membrane abundance in ASAP2-high cells after HGF stimulation, but there were markedly reduced membrane levels after ASAP2 was knocked down (Additional file 1: Fig. S8A). These data indicate that ASAP2 possibly sustains c-MET signaling activation by antagonizing internalization and subsequent degradation of activated c-MET.

ASAP2 mainly exerts its biological function by interacting with other proteins. Therefore, we analyzed potential interactors of ASAP2 using two public databases, BioGRID and HIPPIE (HIPPIE Score > 0.70). Eleven proteins overlapped between these two databases. Interestingly, SH3KBP1, a protein also known as CIN85 that is a critical regulator for inducing c-MET internalization and lysosomal degradation after HGF stimulation, was ranked first according to the HIPPIE database (Fig. 7D). Additionally, the STRING database demonstrated a direct association between ASAP2 and CIN85, but no interaction between ASAP2 and c-MET was predicted (Additional file 1: Fig. S8B). These findings prompted us to hypothesize that ASAP2 potentially interacts with CIN85, inhibiting the recognition and binding of CIN85 to activated c-MET, thus blocking the degradation of c-MET upon HGF stimulation. To validate this, we first co-transfected HA-tagged CIN85 and Flag-tagged ASAP2 into 293 T cells, followed by co-IP and western blot assays. Our results confirmed that ASAP2 could directly interact with CIN85 (Additional file 1: Fig. S8C). Moreover, endogenous interactions between ASAP2 and CIN85 were observed in HCC cells (Fig. 7E), and exogenous ASAP2 could also bind to endogenous CIN85 (Additional file 1: Fig. S8D). Next, the impact of ASAP2 knockdown on the binding of CIN85 to c-MET was evaluated. ASAP2 knockdown led to a strongly enhanced interaction between CIN85 and c-MET (Fig. 7F), while ectopic ASAP2 expression attenuated this interaction (Additional file 1: Fig. S8E). Importantly, ASAP2 could compete with c-MET to bind to CIN85 in a dose-dependent manner (Fig. 7G). Collectively, our data demonstrate the crucial role of ASAP2 in preventing c-MET lysosome-mediated degradation induced by CIN85 by disrupting the CIN85-c-MET interaction.

### Silencing CIN85 alleviates the inhibitory effects of ASAP2 knockdown

Lastly, we validated the essential role of CIN85 in ASAP2-reuglated c-MET activation and HCC progression. Kaplan–Meier analysis indicated that high CIN85 levels served as a protective indicator for OS (Additional file 1: Fig. S9A), RFS (Additional file 1: Fig. S9B), PFS (Additional file 1: Fig. S9C), and disease-specific survival (DSS, Additional file 1: Fig. S9D) in the entire TCGA LIHC cohort. Similarly, hepatitis HCC patients with high CIN85 levels also had significantly longer OS, RFS, PFS, and DSS in TCGA dataset (Additional file 1: Fig. S9E–H), suggesting a protective role of CIN85 in HCC. We next applied siRNAs to interfere with CIN85 expression in ASAP2-knockdown HCC cells. CCK8 experiments indicated that the impaired proliferation potential resulting from ASAP2 knockdown could be almost rescued by silencing CIN85 (Fig. 7H). Consistently, Transwell assays demonstrated that CIN85 expression interference could attenuate the inhibitory effects of ASAP2 knockdown on HCC invasion (Fig. 7I). Critically, siCIN85 could restore c-MET protein stability in ASAP2-knockdown HCC cells, resulting in the reactivation of AKT and ERK1/2 signaling when treated with HGF (Fig. 7J). Additionally, the impaired mesenchymal-like phenotype was recovered by CIN85 interference in ASAP2-knockdwon HCC cells according to qRT-PCR experiments (Additional file 1: Fig. S10). Overall, these data strongly imply that CIN85 is a key target for ASAP2 to promote HCC.

## Discussion

An increased invasion capacity is a main intrinsic force for HCC recurrence, which is a major factor contributing to therapy failure in HCC patients [28]. Hence, elucidating the detailed mechanism regarding increased invasiveness in HCC is critical for improving efficiency of HCC management. Here, we identified ASAP2 as a novel regulator for promoting HCC growth and invasion. Notably, ASAP2 was essential for active c-MET-induced malignant phenotypes, such as EMT, increased growth potential, and enhanced invasiveness in HCC. ASAP2 could effectively antagonize CIN85-induced c-MET internalization and lysosomal degradation by directly binding to CIN85, blocking the interaction between CIN85 and c-MET. HCC cells are essentially locked in a constant “c-MET signaling on” state, which facilitates EMT and recurrence formation.

EMT is a vital driver of HCC invasion [29]. It is a complicated process characterized by the emergence of a mesenchymal-like molecular phenotype with the loss of apical-basal polarity, which renders HCC cells with increased mobility and invasion potential [30]. This transformation allows HCC cells to invade the vascular system and disseminate to intra- or extra-hepatic tissue, eventually inducing recurrence or metastasis [31]. Notably, c-MET signaling was considered one of the major mechanisms for EMT occurrence in HCC [32]. Because c-MET DNA amplification was relatively rare in HCC samples (8/348 of HCC patients in TCGA dataset according to cBioPortal dataset), other unrevealed mechanisms likely help sustain c-MET activation. Unfortunately, few investigations have concurrently clarified this issue. From in vitro and in vivo experiments and clinical sample analysis, we identified ASAP2 as a novel inducer of mesenchymal phenotype transformation in HCC cells. More importantly, we demonstrated that ASAP2 is essential for the long-term activation of c-MET signaling to promote EMT in HCC. Additionally, knockdown of ASAP2 could abolish HGF-induced HCC cell proliferation, invasion, and EMT. Our results suggest a novel regulatory mechanism contributing to the high protein expression levels of c-MET in HCC, implicating ASAP2 as a potent therapeutic target for treating c-MET-activated HCC.

Generally, c-MET signaling is meticulously regulated by CIN85 [22]. Upon stimulation with HGF, c-MET forms a homodimer that undergoes autophosphorylation to generate active signaling in cells. Simultaneously, c-MET also rapidly recruits c-CBL, resulting in ubiquitination of c-MET [33]. Previous research has reported that CIN85 can recognize and bind to ubiquitinated c-MET, triggering subsequent internalization and lysosome-mediated degradation of activated c-MET [22]. Therefore, CIN85 is essential for maintaining the intracellular homeostasis of c-MET and ultimately determines the activation degree and duration of c-MET signaling. However, how CIN85 is involved in the aberrant activation of c-MET in HCC remained unclear. Here, we found a close interaction between ASAP2 and CIN85. ASAP2 could effectively disrupt the binding of CIN85 to activated c-MET, resulting in enhanced c-MET protein stability and prolonged duration of activated c-MET on the cell membrane. Interestingly, high CIN85 expression levels indicated better prognosis for HCC patients, suggesting its protective role during HCC progression. Moreover, interfering with CIN85 expression in ASAP2-knockdown HCC cells could almost restore tumor growth and invasion potentials, validating its role in ASAP2-induced c-MET activation. Our results were consistent with those of previous studies to indicate a negative regulatory role of CIN85 in c-MET signaling [22]. More importantly, our data demonstrate a novel ASAP2-dependent mechanism to sustain long-term c-MET activation to promote EMT and recurrence/metastasis. ASAP2 expression is significantly increased in HCC samples and c-MET signaling remains crucial for certain tissues and cells to perform their normal functions. Therefore, our data imply that targeting tumoral ASAP2 is potentially a more precise therapeutic strategy for inhibiting c-MET signaling in HCC with a minimal impact on c-MET activation in normal cells.

There are several limitations to the present study. First, a detailed mechanism of why ASAP2 exhibited more affinity to CIN85 than activated c-MET requires further exploration. Second, a critical domain or region for the interaction between ASAP2 and CIN85 also remains unclear. Third, whether a reported ASAP2 inhibitor [17], niclosamide, exhibits any anti-tumor effects in HCC needs to be investigated. We will design appropriate experiments aiming to address these issues in the future. Moreover, the prognostic significance of ASAP2 should be validated with HCC patients with different etiological backgrounds, such as Hepatits C (HCV) and nonalcoholic steatohepatitis (NASH). Because ASAP2 DNA copy number was increased in HCC, detecting ASAP2 DNA copy number variations via fluorescent in situ hybridization (FISH) assays could possibly provide useful information for predicting HCC patient prognosis. We also aim to establish an accurate FISH detection system to validate this issue.

## Conclusions

In summary, this study identified ASAP2 as a novel promoter of HCC progression via sustaining c-MET signaling activation. Notably, we revealed a novel competitive mechanism of ASAP2-induced activated c-MET stability enhancement, which resulted in reduced c-MET internalization, degradation, and prolonged intracellular c-MET signal transduction. Our present work might provide novel insight into exploring new therapeutic approaches for HCC patients with aberrant c-MET activation.

## Supplementary Information

**Additional file 1: Figure S1.** Expression pattern of ASAP2 across different types of normal tissues and cancers. (A) ASAP2 expression across different types of normal tissues according to GTEx dataset. (B) ASAP2 expression across different types of normal tissues according to HPA dataset. (C) ASAP2 expression across different types of normal tissues according to FANTOM5 dataset. (D) ASAP2 expression across different types of cancer according to TCGA dataset. **Figure S2.** Correlation between ASAP2 and prognosis and clinicopathological parameters. (A) GSEA results showed WOO Liver Cancer Recurrence UP signature was positively enriched in ASAP2-high HCC, whereas LEE Liver Cancer Survival UP signature was negative associated with ASAP2-high HCC. (B) Heatmap of the correlations between ASAP2 and clinicopathological parameters in FUSCC cohort. **Figure S3.** Prognostic value of ASAP2 across various types of cancer. (A) Prognostic value of ASAP2 for overall survival (OS, upper) and recurrence-free survival (RFS, lower) in cervical squamous cell carcinoma according to TCGA dataset. (B) Prognostic value of ASAP2 for OS (upper) and RFS (lower) in lung adenocarcinoma according to TCGA dataset. (C) Prognostic value of ASAP2 for OS (upper) and RFS (lower) in pancreatic ductal adenocarcinoma according to TCGA dataset. (D) Prognostic value of ASAP2 for OS (upper) and RFS (lower) in uterine corpus endometrial carcinoma according to TCGA dataset. **Figure S4.** Correlations between ASAP2 and proliferation-related molecular signature in HCC. (A) Correlations between ASAP2 and proliferation-related molecular signatures according to TCGA dataset. (B) Correlations between ASAP2 and cell cycle-related molecular signatures according to TCGA dataset. (C) Correlations between ASAP2 and apoptosis-related molecular signatures according to TCGA dataset. (D) Correlations between ASAP2 and proliferation/cell cycle-related markers in TCGA LIHC cohort according to GEPIA dataset. (E) Correlations between ASAP2 and apoptosis-related markers in TCGA LIHC cohort according to GEPIA dataset. **Figure S5.** Associations between ASAP2 and EMT process in HCC. (A) Effects of ASAP2 knockdown on the mRNA expressions of EMT-related markers in SNU182 (upper panel) and SNU387 (lower panel) cells were determined by RT-PCR. (B) Effects of ASAP2 overexpression on the mRNA expressions of EMT-related markers in Hep3B cells were determined by RT-PCR. (C) mRNA expressions of EMT-related marker in HCC tissues derived from indicated mice models were quantified by RT-PCR. (D) Associations between ASAP2 and indicated EMT-related markers according in TCGA LIHC cohort according to GEPIA dataset. “*” indicated 0.01 ≤ P < 0.05; “**” indicated 0.001 ≤ P < 0.01; “****” indicated P < 0.001. **Figure S6.** ASAP2 is required for HGF/c-MET signaling-induced malignant phenotype. (A) GSEA results showed active downstream targets of c-MET signaling were significantly enriched in ASAP2-high HCC. (B) shControl or ASAP2-konckdown SNU182 (upper)/SNU387 (lower) cells were seeded into a Transwell chamber inserts (8 μm pore size, upper chamber) at a density of 5 X 10^4^ cells per well; Up chamber was supplemented with DMEM containing 1% FBS, while the lower chamber was supplemented with DMEM containing 1% FBS with or without HGF (20 ng/ml); Cells were cultured for 24 h to allow migration; Afterwards, upper chambers were collected and the invading cells were quantified by crystal violet staining to evaluate the influence of ASAP2 knockdown on migration potential of HCC cells upon HGF stimulation. (C) Control or ASAP2-overexpressed Hep3B cells were serum-free starved for 12 h; Afterwards, culture medium was replaced by serum-free DMEM containing 1% FBS with or without HGF (20 ng/ml) and cells were culture for 3 days; At day 3, CCK8 assays were conducted to evaluate the effects of ASAP2 overexpression on the proliferation potentials of Hep3B cells upon HGF stimulation. **Figure S7.** ASAP2 promotes Gab1 binding to c-MET, and subsequent phosphorylation in HCC. Effects of ASAP2 knockdown (left) or ASAP2 overexpression (right) on the interaction between c-MET and Gab1, and p-Gab1 level in HCC cells were evaluated by IP and WB assays. **Figure S8.** ASAP2 interrupts CIN85-c-MET interaction. (A) shControl and ASAP2-knockdown SNU182 (left) and SNU387 (right) cells were serum-free starved for 12 h, then culture medium was replaced with fresh serum-free DMEM with or without HGF (20 ng/ml) plus CHX (50 μg/ml); protein was extracted after 2 h treatment, followed by membrane fraction separation; WB assays were further conducted to detect the c-MET abundance on the membrane. (B) Protein–protein interaction network among c-MET, c-CBL, CIN85 and ASAP2 according to STRING database. (C) Interaction between exogenous, HA-tagged CIN85 and Flag-tagged ASPA2 in 293 T cells was confirmed by co-IP and WB assays. (D) Interaction between exogenous ASAP2 and endogenous CIN85 was validated by co-IP and WB assays. (E) Effects of ASAP2 overexpression on the interaction between CIN85 and c-MET were evaluated by IP followed by WB assays; Bafilomycin A1 (BA) was applied to minimize c-MET lysosomal degradation. **Figure S9.** Prognostic value of CIN85 in HCC. (A-D) Prognostic value of CIN85 for predicting OS (A), RFS (B), progression-free survival (PFS, C) and DSS (Disease specific survival, D) in entire TCGA LIHA cohort. (E–H) Prognostic value of CIN85 for predicting OS (E), RFS (F), PFS (G) and DSS (H) in HCC patients with Hepatitis according to TCGA dataset. **Figure S10.** Effects of silencing CIN85 on expressions of EMT-related markers such as Fibronectin, N-Cadherin, E-Cadherin and Vimentin in ASAP2 knockdown SNU182 cells were determined by RT-PCR assay.

**Additional file 2: Table S1.** Primers used for RT-PCR assays. **Table S2.** Antibodies used for WB, IHC and IP assays. **Table S3.** shRNA and siRNA used for expression interference.

## Data Availability

The datasets used and/or analyzed during the current study are available from the corresponding author on reasonable request.
