# Supplementary material for: ASAP2 interrupts c-MET-CIN85 interaction to sustain HGF/c-MET-induced malignant potentials in hepatocellular carcinoma
Source: Exp Hematol Oncol. 2023 Apr 15;12:38. doi: 10.1186/s40164-023-00393-3 (PMC10105420; doi:10.1186/s40164-023-00393-3)
Supplement: Supplementary file 1 — Additional file 1: Figure S1. Expression pattern of ASAP2 across different types of normal tissues and cancers. (A) ASAP2 expression across different types of normal tissues according to GTEx dataset. (B) ASAP2 expression across different types of normal tissues according to HPA dataset. (C) ASAP2 expression across different types of normal tissues according to FANTOM5 dataset. (D) ASAP2 expression across different types of cancer according to TCGA dataset. Figure S2. Correlation between ASAP2 and prognosis and clinicopathological parameters. (A) GSEA results showed WOO Liver Cancer Recurrence UP signature was positively enriched in ASAP2-high HCC, whereas LEE Liver Cancer Survival UP signature was negative associated with ASAP2-high HCC. (B) Heatmap of the correlations between ASAP2 and clinicopathological parameters in FUSCC cohort. Figure S3. Prognostic value of ASAP2 across various types of cancer. (A) Prognostic value of ASAP2 for overall survival (OS, upper) and recurrence-free survival (RFS, lower) in cervical squamous cell carcinoma according to TCGA dataset. (B) Prognostic value of ASAP2 for OS (upper) and RFS (lower) in lung adenocarcinoma according to TCGA dataset. (C) Prognostic value of ASAP2 for OS (upper) and RFS (lower) in pancreatic ductal adenocarcinoma according to TCGA dataset. (D) Prognostic value of ASAP2 for OS (upper) and RFS (lower) in uterine corpus endometrial carcinoma according to TCGA dataset. Figure S4. Correlations between ASAP2 and proliferation-related molecular signature in HCC. (A) Correlations between ASAP2 and proliferation-related molecular signatures according to TCGA dataset. (B) Correlations between ASAP2 and cell cycle-related molecular signatures according to TCGA dataset. (C) Correlations between ASAP2 and apoptosis-related molecular signatures according to TCGA dataset. (D) Correlations between ASAP2 and proliferation/cell cycle-related markers in TCGA LIHC cohort according to GEPIA dataset. (E) Correlations between ASAP [file 40164_2023_393_MOESM1_ESM.docx]

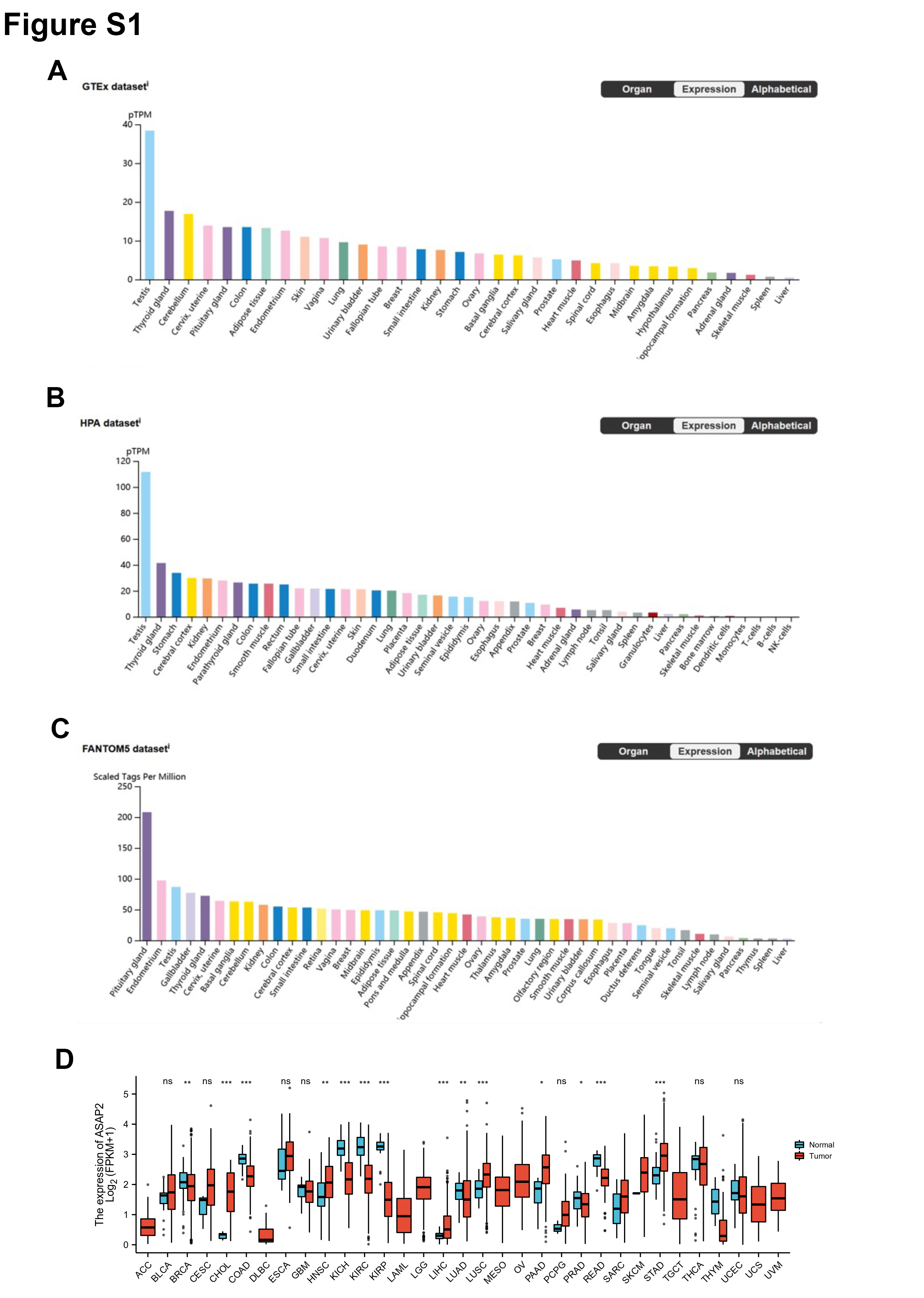
 **Figure S1. Expression pattern of ASAP2 across different types of normal tissues and cancers.** (A) ASAP2 expression across different types of normal tissues according to GTEx dataset. (B) ASAP2 expression across different types of normal tissues according to HPA dataset. (C) ASAP2 expression across different types of normal tissues according to FANTOM5 dataset. (D) ASAP2 expression across different types of cancer according to TCGA dataset.


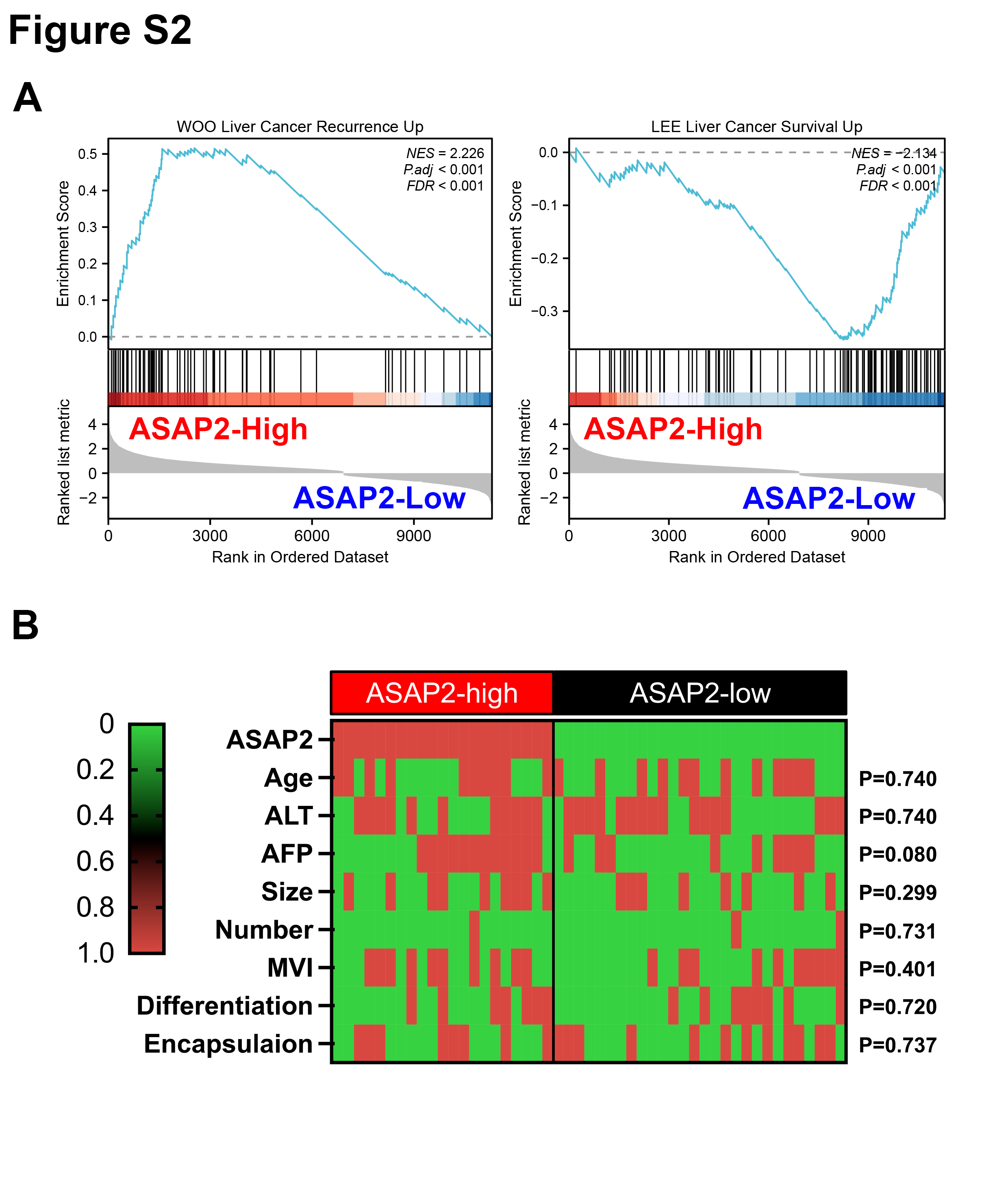
 **Figure S2. Correlation between ASAP2 and prognosis and clinicopathological parameters.** (A) GSEA results showed WOO Liver Cancer Recurrence UP signature was positively enriched in ASAP2-high HCC, whereas LEE Liver Cancer Survival UP signature was negative associated with ASAP2-high HCC. (B) Heatmap of the correlations between ASAP2 and clinicopathological parameters in FUSCC cohort.

**
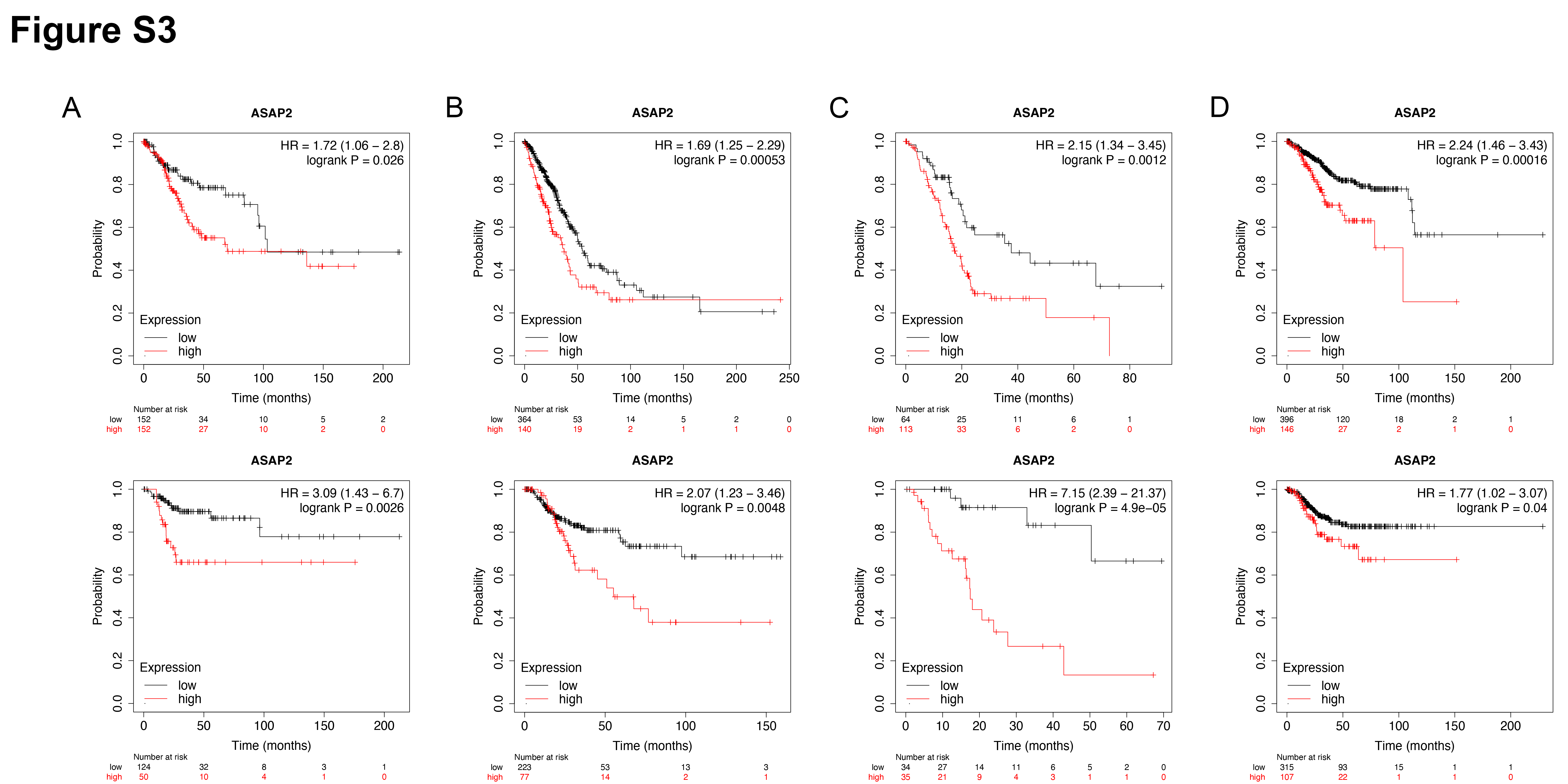
**

**Figure S3. Prognostic value of ASAP2 across various types of cancer.** (A) Prognostic value of ASAP2 for overall survival (OS, upper) and recurrence-free survival (RFS, lower) in cervical squamous cell carcinoma according to TCGA dataset. (B) Prognostic value of ASAP2 for OS (upper) and RFS (lower) in lung adenocarcinoma according to TCGA dataset. (C) Prognostic value of ASAP2 for OS (upper) and RFS (lower) in pancreatic ductal adenocarcinoma according to TCGA dataset. (D) Prognostic value of ASAP2 for OS (upper) and RFS (lower) in uterine corpus endometrial carcinoma according to TCGA dataset.

**
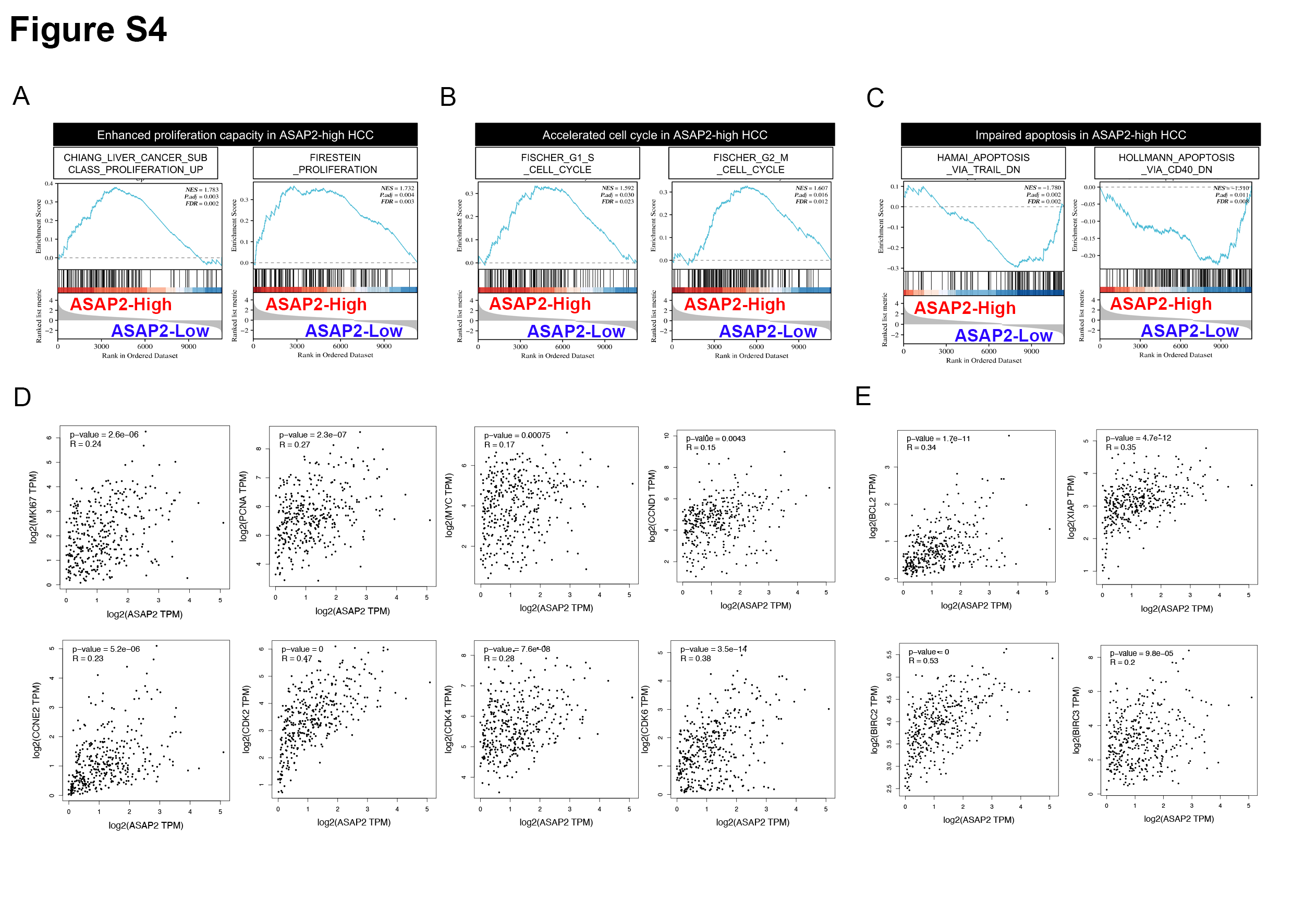
**

**Figure S4. Correlations between ASAP2 and proliferation-related molecular signature in HCC.** (A) Correlations between ASAP2 and proliferation-related molecular signatures according to TCGA dataset. (B) Correlations between ASAP2 and cell cycle-related molecular signatures according to TCGA dataset. (C) Correlations between ASAP2 and apoptosis-related molecular signatures according to TCGA dataset. (D) Correlations between ASAP2 and proliferation/cell cycle-related markers in TCGA LIHC cohort according to GEPIA dataset. (E) Correlations between ASAP2 and apoptosis-related markers in TCGA LIHC cohort according to GEPIA dataset.


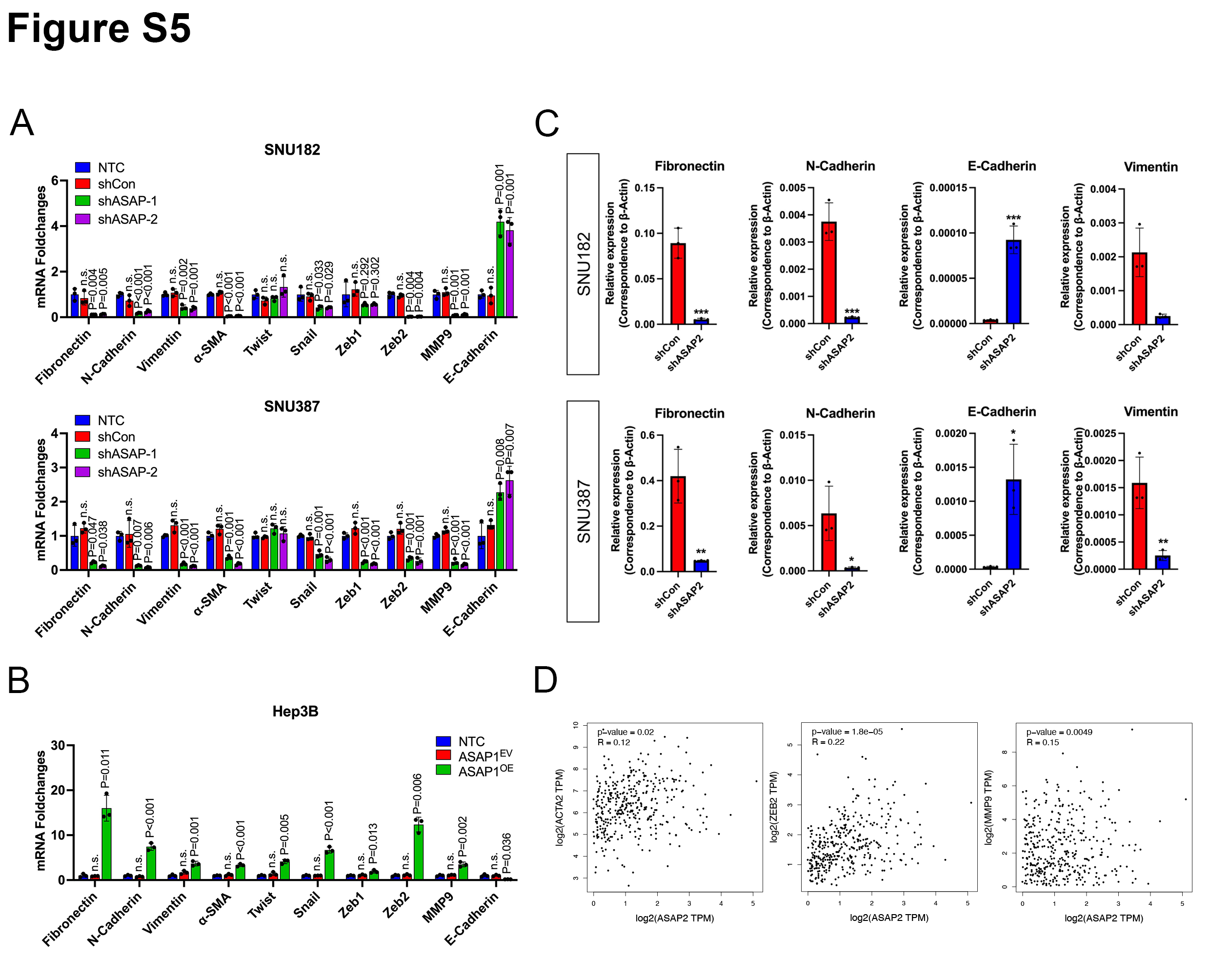
 **Figure S5. Associations between ASAP2 and EMT process in HCC.** (A) Effects of ASAP2 knockdown on the mRNA expressions of EMT-related markers in SNU182 (upper panel) and SNU387 (lower panel) cells were determined by RT-PCR. (B) Effects of ASAP2 overexpression on the mRNA expressions of EMT-related markers in Hep3B cells were determined by RT-PCR. (C) mRNA expressions of EMT-related marker in HCC tissues derived from indicated mice models were quantified by RT-PCR. (D) Associations between ASAP2 and indicated EMT-related markers according in TCGA LIHC cohort according to GEPIA dataset. “*” indicated 0.01≤P<0.05; “**” indicated 0.001≤P<0.01; “****” indicated P<0.001.


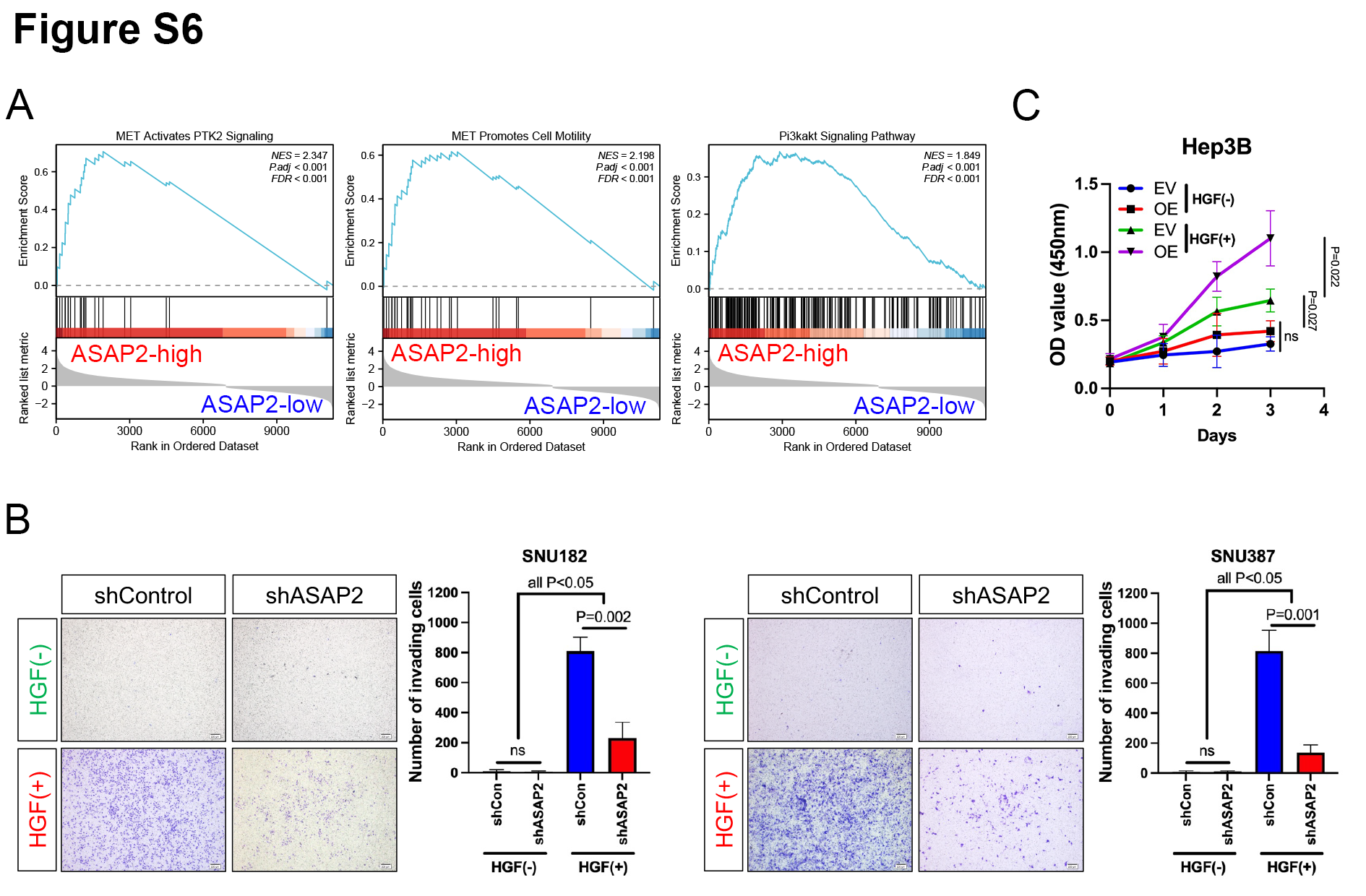
 **Figure S6. ASAP2 is required for HGF/c-MET signaling-induced malignant phenotype.** (A) GSEA results showed active downstream targets of c-MET signaling were significantly enriched in ASAP2-high HCC. (B) shControl or ASAP2-konckdown SNU182 (upper)/SNU387 (lower) cells were seeded into a Transwell chamber inserts (8μm pore size, upper chamber) at a density of 5 X 10^4^ cells per well; Up chamber was supplemented with DMEM containing 1% FBS, while the lower chamber was supplemented with DMEM containing 1% FBS with or without HGF (20 ng/ml); Cells were cultured for 24 hours to allow migration; Afterwards, upper chambers were collected and the invading cells were quantified by crystal violet staining to evaluate the influence of ASAP2 knockdown on migration potential of HCC cells upon HGF stimulation. (C) Control or ASAP2-overexpressed Hep3B cells were serum-free starved for 12 hours; Afterwards, culture medium was replaced by serum-free DMEM containing 1% FBS with or without HGF (20 ng/ml) and cells were culture for 3 days; At day 3, CCK8 assays were conducted to evaluate the effects of ASAP2 overexpression on the proliferation potentials of Hep3B cells upon HGF stimulation.


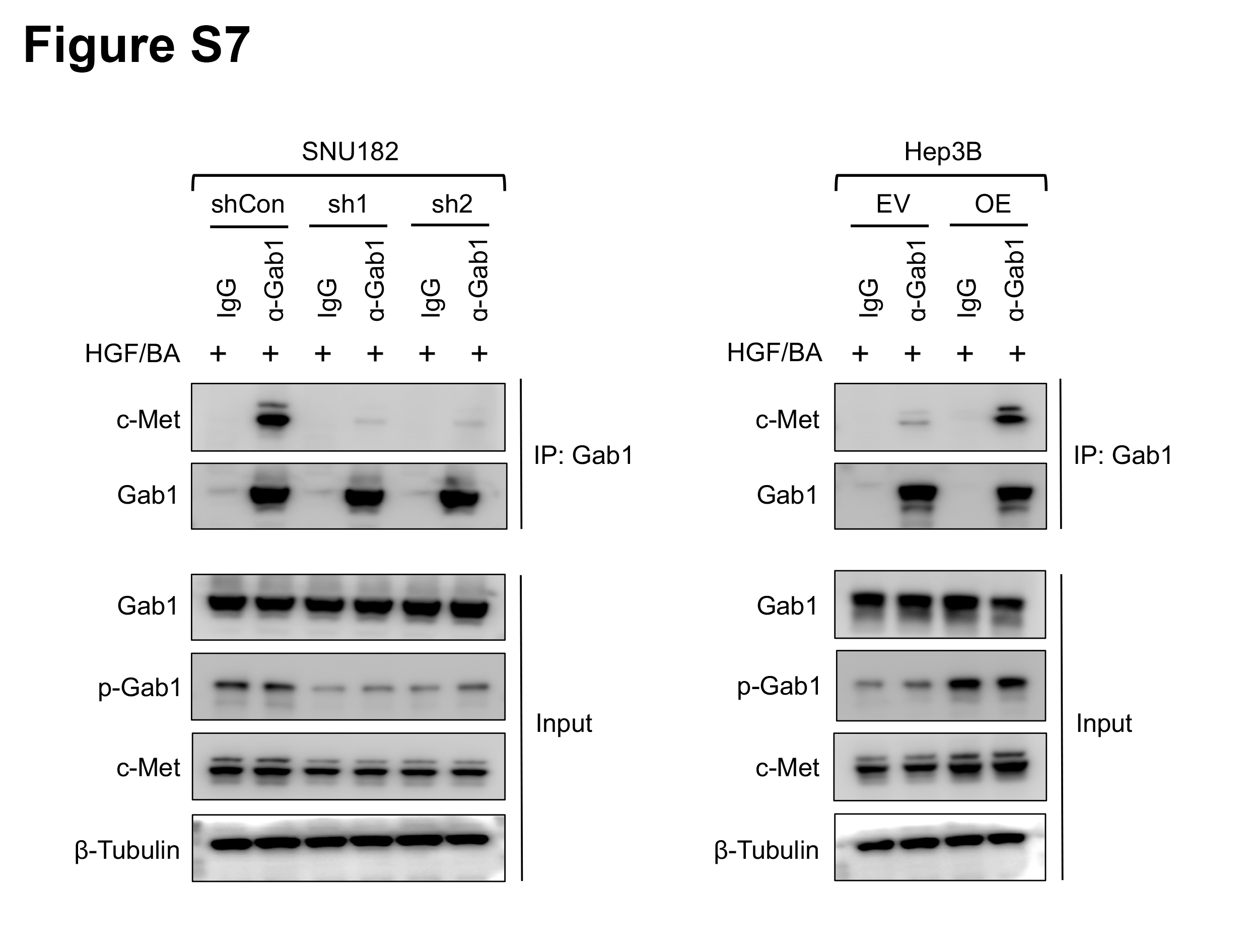
 **Figure S7. ASAP2 promotes Gab1 binding to c-MET, and subsequent phosphorylation in HCC.** Effects of ASAP2 knockdown (left) or ASAP2 overexpression (right) on the interaction between c-MET and Gab1, and p-Gab1 level in HCC cells were evaluated by IP and WB assays.


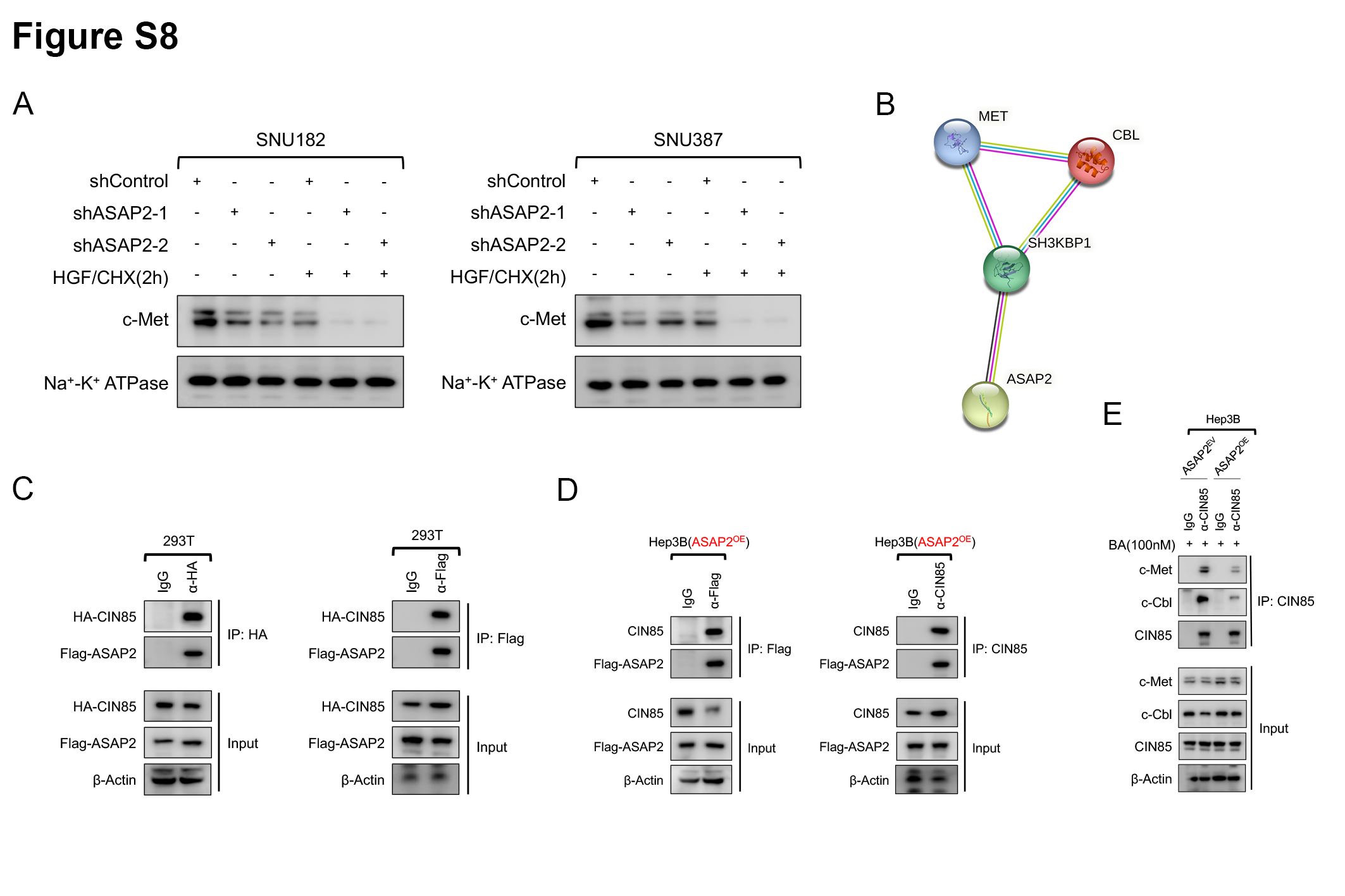
 **Figure S8. ASAP2 interrupts CIN85-c-MET interaction.** (A) shControl and ASAP2-knockdown SNU182 (left) and SNU387 (right) cells were serum-free starved for 12 hours, then culture medium was replaced with fresh serum-free DMEM with or without HGF (20 ng/ml) plus CHX (50 μg/ml); protein was extracted after 2 hours treatment, followed by membrane fraction separation; WB assays were further conducted to detect the c-MET abundance on the membrane. (B) Protein-protein interaction network among c-MET, c-CBL, CIN85 and ASAP2 according to STRING database. (C) Interaction between exogenous, HA-tagged CIN85 and Flag-tagged ASPA2 in 293T cells was confirmed by co-IP and WB assays. (D) Interaction between exogenous ASAP2 and endogenous CIN85 was validated by co-IP and WB assays. (E) Effects of ASAP2 overexpression on the interaction between CIN85 and c-MET were evaluated by IP followed by WB assays; Bafilomycin A1 (BA) was applied to minimize c-MET lysosomal degradation.


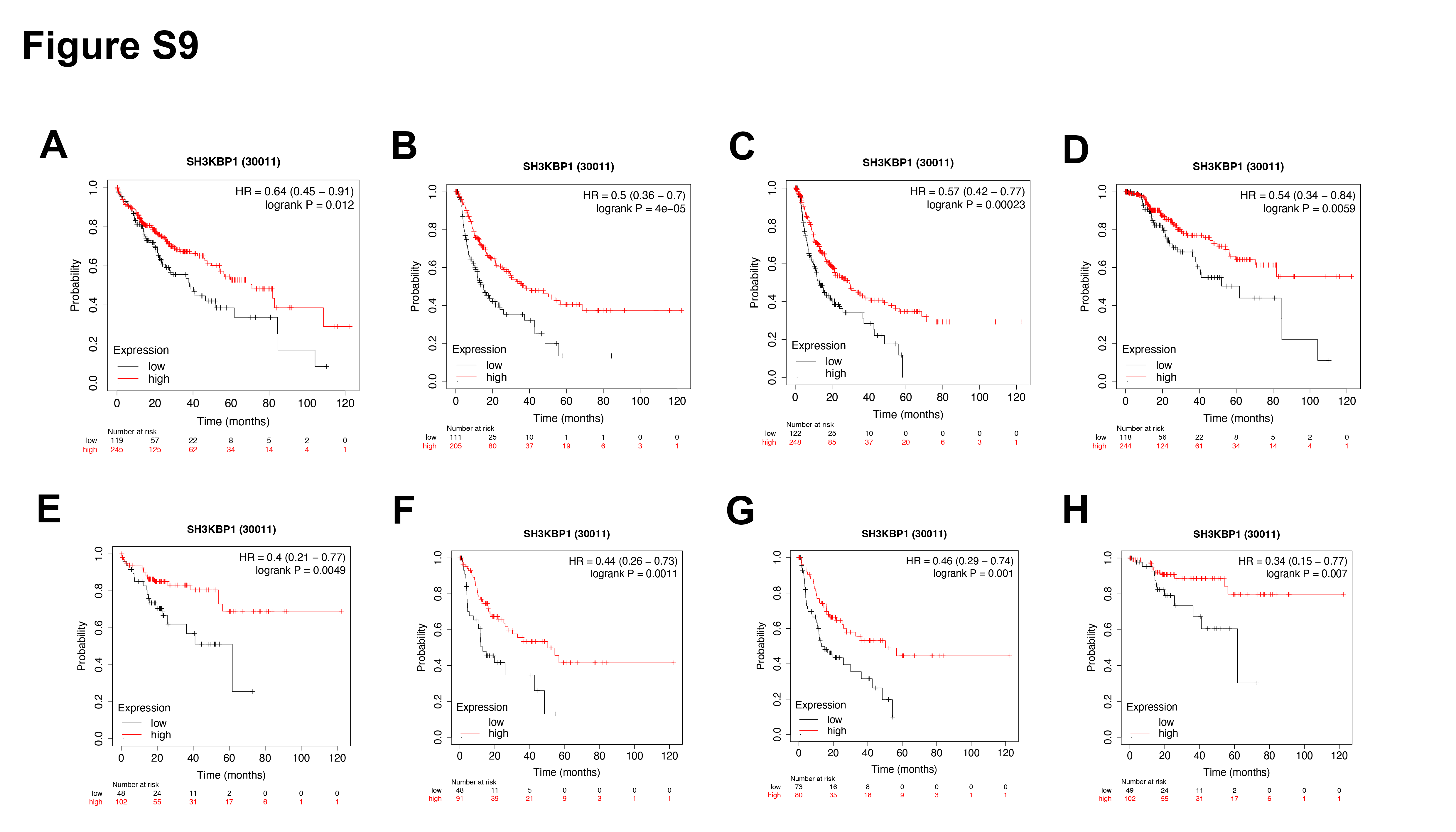
 **Figure S9. Prognostic value of CIN85 in HCC.** (A-D) Prognostic value of CIN85 for predicting OS (A), RFS (B), progression-free survival (PFS, C) and DSS (Disease specific survival, D) in entire TCGA LIHA cohort. (E-H) Prognostic value of CIN85 for predicting OS (E), RFS (F), PFS (G) and DSS (H) in HCC patients with Hepatitis according to TCGA dataset.


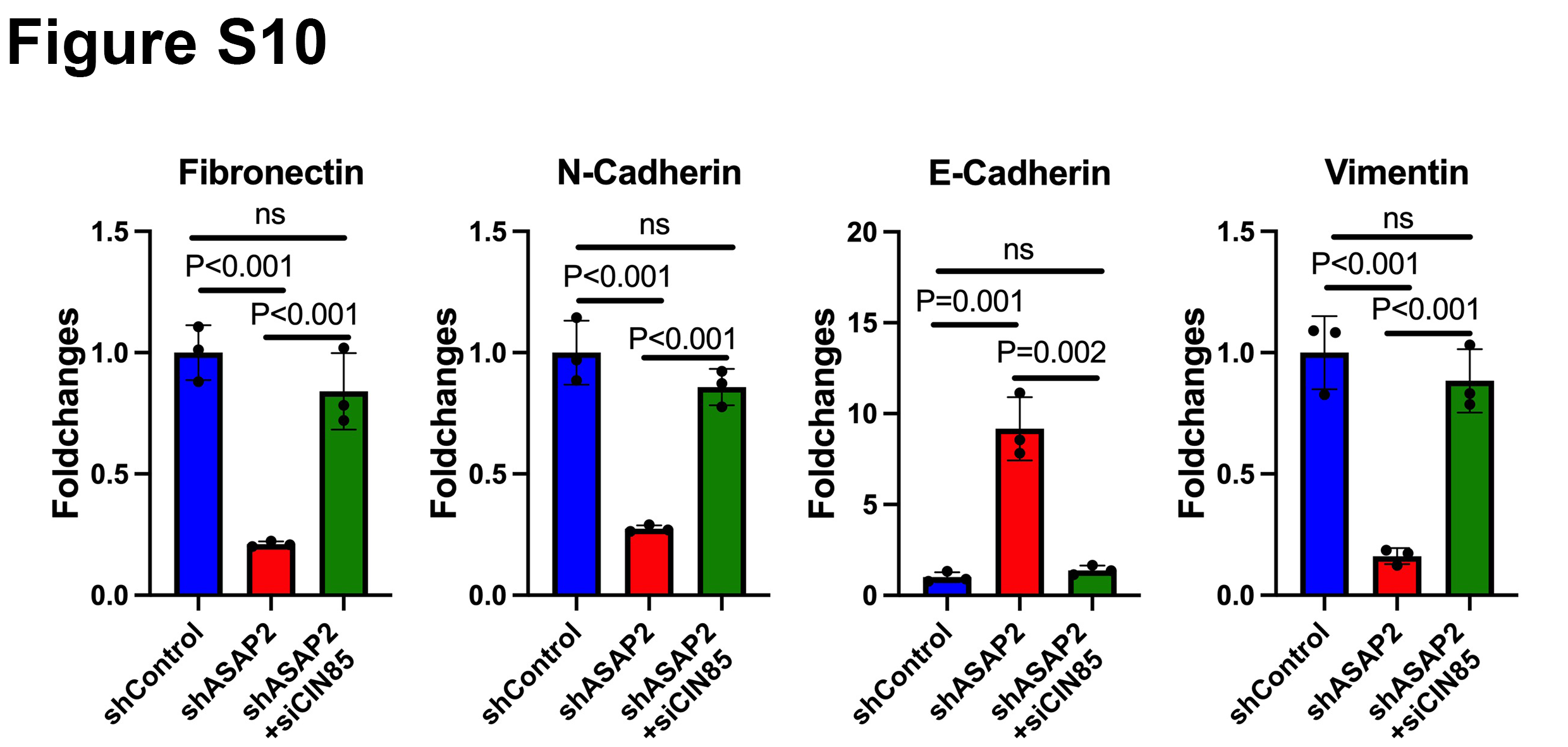


**Figure S10. Effects of silencing CIN85 on expressions of EMT-related markers such as Fibronectin, N-Cadherin, E-Cadherin and Vimentin in ASAP2 knockdown SNU182 cells were determined by RT-PCR assay.**
