# Supplementary material for: ASAP2 interrupts c-MET-CIN85 interaction to sustain HGF/c-MET-induced malignant potentials in hepatocellular carcinoma
Source: Exp Hematol Oncol. 2023 Apr 15;12:38. doi: 10.1186/s40164-023-00393-3 (PMC10105420; doi:10.1186/s40164-023-00393-3)
Supplement: Supplementary file 2 — Additional file 2: Table S1. Primers used for RT-PCR assays. Table S2. Antibodies used for WB, IHC and IP assays. Table S3. shRNA and siRNA used for expression interference. [file 40164_2023_393_MOESM2_ESM.docx]

| Table S1. Primers used for RT-PCR assays | | |
| --- | --- | --- |
|  | Forward (5’->3’) | Backward (5’->3’) |
| ASAP2 | AATAAGCGGAGCGGAAATTGC | GTTTCAATGGAAGGTTTGAGGC |
| Fibronectin | AGCCGAGGTTTTAACTGCGA | CCCACTCGGTAAGTGTTCCC |
| CDH2 | AGCCAACCTTAACTGAGGAGT | GGCAAGTTGATTGGAGGGATG |
| Vimentin | TGCCGTTGAAGCTGCTAACTA | CCAGAGGGAGTGAATCCAGATTA |
| α-SMA | AAAAGACAGCTACGTGGGTGA | GCCATGTTCTATCGGGTACTTC |
| Twist | GTCCGCAGTCTTACGAGGAG | GCTTGAGGGTCTGAATCTTGCT |
| Snail | ACTGCAACAAGGAATACCTCAG | GCACTGGTACTTCTTGACATCTG |
| ZEB1 | GCAGTCCAAGAACCACCCTT | GGGCGGTGTAGAATCAGAGT |
| ZEB2 | GCTAGTGTGCCCAACCATGA | AGTCTTCCTTCATTTCTTCTGGACC |
| MMP9 | GGGACGCAGACATCGTCATC | TCGTCATCGTCGAAATGGGC |
| CDH1 | AAAGGCCCATTTCCTAAAAACCT | TGCGTTCTCTATCCAGAGGCT |
| c-MET | TGTCCACAGAGACTTGGCTG | CGCCAAAGGACCACACATCT |
| β-Actin | CATGTACGTTGCTATCCAGGC | CTCCTTAATGTCACGCACGAT |

| Table S2. Antibodies used for WB, IHC and IP assays | | |
| --- | --- | --- |
| Target | Brand | Use (dilution) |
| ASAP2 | Santa Cruz | WB (1:1000) |
|  |  | IHC (1:100) |
|  |  | IP (1:25) |
| CCNE | CST | WB (1:1000) |
| PCNA | CST | WB (1:1000) |
| BCL2 | CST | WB (1:1000) |
| c-Caspase 3 | CST | WB (1:1000) |
| Fibronectin | Abcam | WB (1:1000) |
| N-Cadherin | CST | WB (1:1000) |
|  |  | IHC (1:100) |
| E-Cadherin | CST | WB (1:1000) |
|  |  | IHC (1:100) |
| Vimentin | CST | WB (1:1000) |
| Snail | CST | WB (1:1000) |
| MMP9 | CST | WB (1:1000) |
| ZEB2 | ProteinTech | WB (1:500) |
| c-MET | CST | WB (1:1000) |
|  |  | IHC (1:100) |
| p-AKT | CST | WB (1:1000) |
| AKT | CST | WB (1:1000) |
| p-ERK1/2 | CST | WB (1:1000) |
| Na^+^-K^+^ ATPase | ProteinTech | WB (1:500) |
| HA | CST | WB (1:1000) |
|  |  | IP (1:50) |
| Flag | CST | WB (1:1000) |
|  |  | IP (1:50) |
| CIN85 | Santa Cruz | WB (1:1000) |
|  |  | IP (1:25) |
| c-CBL | CST | WB (1:1000) |
| Gab1 | Abcam | WB (1:1000) |
|  |  | IP (1:25) |
| β-Actin | CST | WB (1:2000) |
| β-Tubulin | CST | WB (1:2000) |

| Table S3. shRNA and siRNA used for expression interference | | |
| --- | --- | --- |
| shRNAs | | |
| Target | Seeded sequence (5’->3’) | |
| shASAP2-1 | GCAGAGATGACCCAGATTTAG | |
| shASAP2-2 | GCGACATGAATGCAAGAAAGG | |
| shASAP2-3 | GCCTCCATCGAGATAGCAAAC | |
| siRNA | | |
| Target | guide (5′→3′) | passenger (5′→3′) |
| CIN85 | ACAAUAACUGUUUAAUCUCUC | GAGAUUAAACAGUUAUUGUCU |
